# Multi-rhythmicity generated by coupling two cellular rhythms

**DOI:** 10.1098/rsif.2018.0835

**Published:** 2019-03-06

**Authors:** Jie Yan, Albert Goldbeter

**Affiliations:** 1 Center for Systems Biology, School of Mathematical Sciences, Soochow University, Suzhou, People's Republic of China; 2 Unité de Chronobiologie Théorique, Faculté des Sciences, Université Libre de Bruxelles (ULB), Brussels, Belgium

**Keywords:** oscillations, biological rhythms, multiple attractors, birhythmicity, trirhythmicity

## Abstract

The cell cycle and the circadian clock represent two major cellular rhythms, which are coupled because the circadian clock governs the synthesis of several proteins of the network that drives the mammalian cell cycle. Analysis of a detailed model for these coupled cellular rhythms previously showed that the cell cycle can be entrained at the circadian period of 24 h, or at a period of 48 h, depending on the autonomous period of the cell cycle and on the coupling strength. We show by means of numerical simulations that multiple stable periodic regimes, i.e. multi-rhythmicity, may originate from the coupling of the two cellular rhythms. In these conditions, the cell cycle can evolve to any one of two (birhythmicity) or three stable periodic regimes (trirhythmicity). When applied at the right phase, transient perturbations of appropriate duration and magnitude can induce the switch between the different oscillatory states. Such switching is characterized by final state sensitivity, which originates from the complex structure of the attraction basins. By providing a novel instance of multi-rhythmicity in a realistic model for the coupling of two major cellular rhythms, the results throw light on the conditions in which multiple stable periodic regimes may coexist in biological systems.

## 1. Introduction

Besides oscillations, spatial patterns and propagating waves, the coexistence between multiple attractors represents a major mode of self-organization in nonlinear systems [1–4]. The coexistence between multiple attractors can take several forms. The most common, known as bistability, pertains to the coexistence between two stable steady states [1–4]. Hard excitation involves the coexistence of a stable steady state with a stable periodic regime, while multirhythmicity involves the coexistence between two (birhythmicity) or three (trirhythmicity) distinct oscillatory states. The present study focuses on the occurrence of multiple periodic attractors in biological systems.

If the phenomenon of bistability, associated with switch-like behaviour, has been observed experimentally in a number of biological systems [2,4–9], it has primarily been observed in a large number of theoretical models pertaining to different modes of biological regulation, from the cellular level up to the level of animal populations [2,4]. Bistable transitions have also been invoked in the aetiology of a number of diseases [4]. Cell differentiation remains, however, the field in which the occurrence of multiple steady states has repeatedly been implicated in the mechanism underlying cell fate specification [4,10–12]. Theoretical models suggest that differentiation into different cell types may sometimes involve tristability, i.e. the coexistence between three stable steady states [13–15]. The coexistence of a stable steady state and a stable periodic regime is also known in a biological context, both experimentally [16] and theoretically [17], but appears to be less common than bistability.

Also less common are examples of birhythmicity, which involves the coexistence between two stable oscillatory regimes. Birhythmicity has been observed in a variety of models for cellular oscillatory processes [18–26], as well as in physical systems [3] for which some theoretical studies aim at controlling the phenomenon to transform it into monorhythmicity [3,27]. Trirhythmicity, i.e. the coexistence between three stable oscillatory regimes, is even more rare than birhythmicity, and has been observed [28] in a three-variable model for two oscillatory enzyme reactions coupled in series [18]. Experimentally, evidence for birhythmic behaviour has been reported for a chemical system involving two coupled oscillatory reactions [29]. Birhythmicity, but not trirhythmicity, has been found in a few biological examples pertaining to neuronal activity. Thus, hysteresis involving two firing frequencies has been observed in cat motor neurons [30]. Similarly, upon perturbation, the R15 neuron of *Aplysia* is capable of switching reversibly between tonic oscillations of the membrane potential and complex oscillations of the bursting type [31]. Because of the scarcity of observations of birhythmicity in biological systems, it is important to delineate the conditions in which multiple periodic attractors may coexist, so as to guide their experimental investigation.

Until now, birhythmicity has mostly been characterized in models for cellular regulatory processes involving multiple sources of oscillations [22]. Thus, the transition between two stable oscillatory regimes was observed in models for two oscillatory enzyme reactions coupled in series [18] or in parallel [21], a product-activated enzyme reaction with substrate recycling [19], cyclic AMP signalling in *Dictyostelium* amoebae based on receptor desensitization [20], the circadian clock network in *Drosophila* [23], the mammalian cell cycle [24,25] and the p53–Mdm2 oscillatory network [26]. In these systems, birhythmicity generally arises from the interplay between two endogenous oscillatory mechanisms. Here, we report a novel mechanism for the occurrence of bi- and trirhythmicity in a realistic model for the coupling between two major cellular rhythms: the circadian clock network producing self-sustained oscillations with a period close to 24 h and the oscillatory biochemical network driving the mammalian cell cycle. These rhythms are coupled because the circadian clock protein BMAL1, which behaves as a transcription factor, controls the expression of several proteins of the cell cycle network.

We previously showed that the control of the cell cycle by the circadian clock can entrain the former to a period of 24 h or 48 h [32]. We now use the model for the coupled system to show, by numerical simulations, that the forcing of the cell cycle by the circadian clock can generate birhythmicity and trirhythmicity. In §2, we briefly describe the model for the coupling of the mammalian cell cycle to the circadian clock. After summarizing the results previously obtained for the effects of this coupling on the dynamics of the cell cycle, such as entrainment and complex oscillations, we investigate the occurrence of birhythmicity and trirhythmicity in §§3 and 4, respectively. We examine in §5 how the system can switch between multiple periodic attractors, before discussing these results in §6.

## 2. Modelling two coupled cellular rhythms: the mammalian cell cycle and the circadian clock

As in a previous study of entrainment of the cell cycle by the circadian clock [32], the model for the coupling of these major cellular rhythms is based on two detailed models proposed for the mammalian cell cycle [33] and the mammalian circadian clock [34,35]. In an appropriate range of parameter values, these two models display sustained oscillatory behaviour of the limit cycle type [33–35].

A network of cyclin-dependent kinases (Cdks) governs the dynamics of the mammalian cell cycle [36]. Different cyclin/Cdk complexes control the transitions between the successive phases of the cell cycle: M (mitosis, or cell division), G1, S (DNA replication phase) and G2. Thus cyclin D/Cdk4–6 and cyclin E/Cdk2 promote progression in G1 and elicit the G1/S transition, respectively; cyclin A/Cdk2 ensures progression in S and the transition S/G2, while the activity of cyclin B/Cdk1 brings about the G2/M transition. A detailed model [33] takes into account the multiple levels of regulation of the Cdk network [36]: cyclin synthesis and degradation, binding of the Cdks to inhibitory proteins such as p21, regulation of cyclin synthesis by the cell cycle inhibitor protein pRb and by its antagonist, the transcription factor E2F, and control of Cdk activity by reversible phosphorylation. This 39-variable model shows that, in the presence of sufficient amounts of growth factor, the Cdk network is capable of temporal self-organization in the form of sustained oscillations, which correspond to the ordered, sequential, transient activation of the various cyclin/Cdk complexes that control the successive phases of the cell cycle [33,37]. A reduced version of the cell cycle model containing less biochemical details but retaining the same regulatory structure displays similar sustained oscillations in Cdk activity, as well as complex oscillations, chaos and birhythmicity [25]. The latter phenomenon has not been observed so far in the more detailed model for the mammalian cell cycle.

The model for the mammalian circadian clock mechanism is based on transcriptional–translational feedback loops involving a limited number of clock proteins. The complex between the proteins CLOCK and BMAL1 induces the expression of the genes coding for the proteins PER and CRY. The complex between these two proteins binds to and inactivates the CLOCK–BMAL1 complex. In addition to this indirect negative feedback exerted by PER and CRY on the expression of their genes, CLOCK–BMAL1 exerts via the protein REV-ERB*α* a negative feedback on BMAL1 expression. The model for the mammalian circadian clock contains 19 variables [34,35].

**Figure 1.**
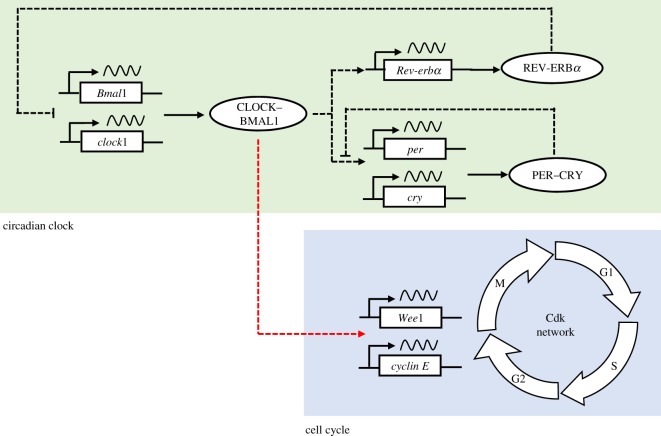
Coupling the cell cycle to the circadian clock. The circadian oscillatory network (in green) controls the cyclin-dependent kinase (Cdk) network (in blue) in various ways, e.g. through regulation (red dashed line) by the circadian clock protein BMAL1 of the expression of cell cycle genes coding for Wee1 (a protein kinase inhibiting Cdks) and cyclin E. Also shown are the main positive and negative feedback loops (black dashed lines) involving, in the circadian clock network, the protein complexes PER–CRY and CLOCK–BMAL1, as well as the protein REV-ERB*α*. Detailed schemes of the models for the cell cycle and the circadian clock are presented in [33,34], respectively. (Online version in colour.)

Coupling of the cell cycle to the circadian clock occurs in multiple ways. Thus, the circadian clock protein BMAL1 regulates the transcription of cell cycle genes coding for (i) the protein kinase Wee1 that inhibits Cdk1 and Cdk2 through reversible phosphorylation [38] and (ii) cyclin E [39,40]. In the model, the coupling of the cell cycle to the circadian clock is introduced by including a BMAL1-dependent rate of mRNA synthesis for Wee1 or cyclin E. Modulating the strength of coupling can be achieved by varying the BMAL1-dependent rate of mRNA synthesis with respect to a basal rate of synthesis (see the electronic supplementary material for the equations that govern the time evolution of the coupled cell cycle–circadian clock system, and for parameter values and initial conditions used in numerical simulations). The coupled cell cycle–circadian clock system is schematized in figure 1.

**Figure 2.**
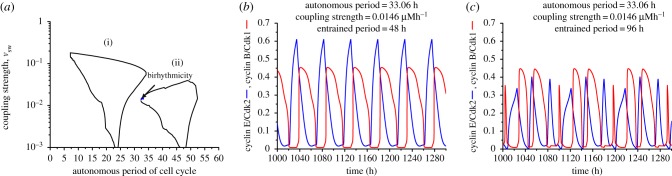
Birhythmicity: coexistence between two stable periodic regimes, in conditions where the cell cycle is coupled to the circadian clock only via Wee1. (*a*) Domains of entrainment of the cell cycle to either 24 h (i) or 48 h (ii), depending on the coupling strength measured by parameter *v*_sw_ and on the autonomous period of the cell cycle prior to its coupling to the circadian clock [32]. The cell cycle is considered to be entrained when Cdk2 and Cdk1, which are variables in the cell cycle network, both exhibit one large-amplitude peak per 24 h or 48 h. Birhythmicity is observed in the small area in blue in the domain where the cell cycle can be entrained to 48 h. (*b,c*) Time evolution of cyclin B/Cdk1 and cyclin E/Cdk2 when the autonomous period of the cell cycle is 33.06 h, and the coupling strength *v*_sw_ is equal to 0.0146 μMh^−1^. The cell cycle is entrained to 48 h (*b*) or exhibits complex oscillations with multiple large peaks in a cycle with a period of 96 h (*c*), depending on the initial conditions. The autonomous period is obtained by setting the value of the scaling parameter *eps* = 13 in (*b*) and (*c*). For this and subsequent figures, see the electronic supplementary material for kinetic equations and parameter values (§§1 and 2), and the initial conditions (§6). As in subsequent figures, the curves in blue and red in (*b*) and (*c*) show the time evolution of cyclin E/Cdk2 and cyclin B/Cdk1, respectively. (Online version in colour.)

**Figure 3.**
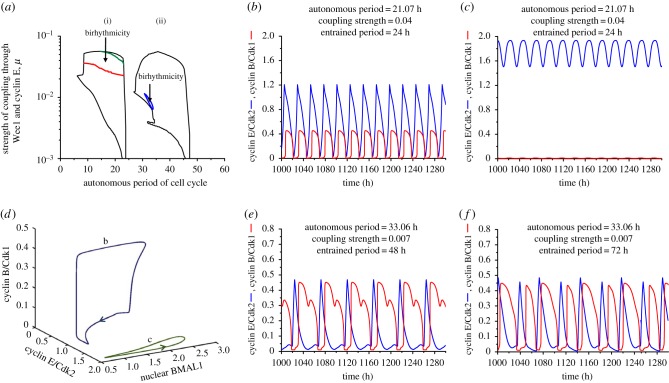
Birhythmicity in conditions where the cell cycle is coupled to the circadian clock via both Wee1 and cyclin E. (*a*) The cell cycle can be entrained to 24 h (i) or 48 h (ii) depending on the coupling strength measured by parameter *μ* and on the autonomous period of the cell cycle prior to its coupling to the circadian clock [32]. Birhythmicity is observed in the area between the green and red lines (i) and in the blue domain (ii). (*b,c*) Time evolution of cyclin B/Cdk1 and cyclin E/Cdk2 when the autonomous period of the cell cycle is 21.07 h, and the coupling strength is *μ* = 0.04. The cell cycle is entrained to 24 h (*b*) or exhibits endoreplication, i.e. oscillations in cyclin E/Cdk2 without large-amplitude oscillations of cyclin B/Cdk1 (*c*), depending on the initial conditions. The autonomous period is obtained in (*b*) and (*c*) by setting the value of the scaling parameter *eps* = 20.4. (*d*) Three-dimensional projections of the two limit cycles corresponding to the coexisting oscillations shown in (*b*) and (*c*). The periodic trajectories followed in phase space by the cell cycle coupled to the circadian clock are projected as a function of cyclin B/Cdk1, cyclin E/Cdk2 and nuclear BMAL1. (*e,f*) Time evolution of cyclin B/Cdk1 and cyclin E/Cdk2 when the autonomous period of the cell cycle is 33.06 h, and the coupling strength is *μ* = 0.007. The cell cycle is entrained to 48 h (*e*) or exhibits complex oscillations with a period of 72 h (*f*), depending on the initial conditions. The autonomous period is obtained in (*e*) and (*f*) by setting the value of the scaling parameter *eps* = 13. (Online version in colour.)

**Figure 4.**
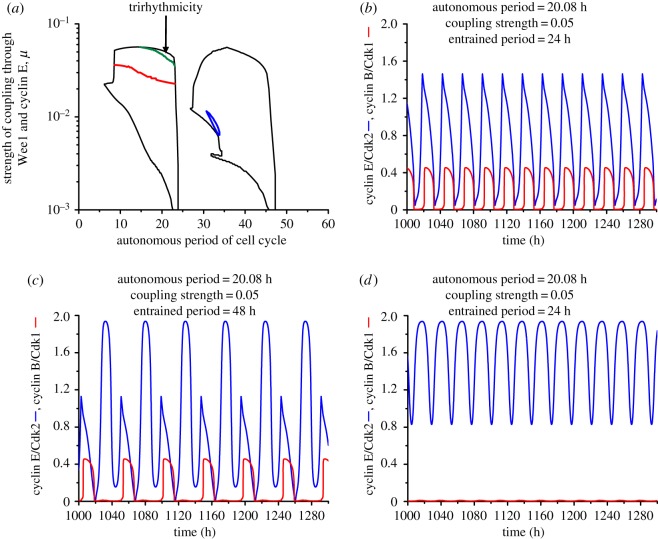
Trirhythmicity: coexistence of three stable periodic regimes, in conditions where the cell cycle is coupled to the circadian clock via both Wee1 and cyclin E. (*a*) Trirhythmicity is observed in the left entrainment domain above the green line, which marks the boundary between the regions of birhythmicity and trirhythmicity. (*b–d*) Time evolutions of cyclin B/Cdk1 and cyclin E/Cdk2 when the autonomous period of the cell cycle is 20.08 h, and the coupling strength is *μ* = 0.05. The cell cycle may be entrained to 24 h (*b*) and exhibits complex oscillation of 48 h (*c*) or endoreplication (*d*), depending on the initial conditions. The autonomous period is obtained by setting the value of the scaling parameter *eps* = 21.4 in (*b–d*). (Online version in colour.)

A detailed study of coupling the cell cycle to the circadian clock was previously performed as a function of the coupling strength and of the period of the cell cycle prior to coupling [32]. The results showed entrainment of the cell cycle by the circadian clock to the period of 24 h or 48 h. As a function of coupling strength and of cell cycle period prior to coupling, the domains of entrainment take the form of Arnold tongues (see [32] and figures 2–4). Complex periodic oscillations, chaos or evolution to a stable steady state were observed in the absence of entrainment [32], but no evidence was found for the coexistence between multiple periodic regimes. Here, we present evidence for the occurrence of birhythmicity and trirhythmicity as a result of the coupling of the cell cycle to the circadian clock.

## 3. Birhythmicity: coexistence of two periodic attractors

The detailed computational models for the cell cycle and the circadian clock contain 41 and 19 variables, respectively, so that the model for the coupled system involves 60 variables. Because of such high dimensionality, regions of coexistence of multiple periodic attractors were identified in parameter space by numerical integration of the kinetic equations (see electronic supplementary material, §§1, 2 and 6). The use of programs for the construction of bifurcation diagrams indeed becomes cumbersome when the number of variables becomes large, as in the present case.

We previously delineated the domains of entrainment of the cell cycle by the circadian clock as a function of coupling strength and of the autonomous period of the cell cycle prior to coupling [32]. Two domains of entrainment were determined by numerical simulations: one in which the cell cycle acquires a period of 24 h, and one in which the cell cycle is entrained to a 48 h period, i.e. twice the forcing period (see [32] and figures 2*a*,3*a*; electronic supplementary material, figures S1a and S2a). In each of these domains, simple periodic oscillations were observed, in which one large-amplitude peak in cyclin E/Cdk2 (associated with the S phase of DNA replication) precedes one large-amplitude peak in cyclin B/Cdk1 (associated with cell division, i.e. mitosis). These two variables play key roles in the cell cycle and were therefore selected to represent the dynamics of the whole Cdk network. The width of the entrainment domains reduces progressively as the coupling strength diminishes, which is a characteristic feature of Arnold tongues.

We explored in further detail the entrainment domains to search for evidence of multiple periodic regimes in the case where the cell cycle is coupled to the circadian clock via Wee1 only, via cyclin E only, or via both Wee1 and cyclin E. Several domains in which birhythmicity occurs could be identified. Evidence for trirhythmicity was also obtained (see §4). We started by considering that the coupling between the cell cycle and the circadian clock occurs only through the induction of Wee1 by BMAL1. In such a case, a small region of birhythmicity was identified at the upper boundary, on the left of the domain of entrainment to 48 h (figure 2*a*). In this relatively minute region (in blue) of the entrainment domain, two stable oscillatory regimes differing in period and amplitude coexist: for certain initial conditions, the system undergoes sustained oscillations with a period of 48 h (figure 2*b*) in which one large-amplitude peak in cyclin B/Cdk1 (red curve) follows one large-amplitude peak in cyclin E/Cdk2 (blue curve). These periodic oscillations are simple (as opposed to complex) because for a given variable all peaks in the time series are identical. By contrast, for some other initial conditions, one large-amplitude peak in cyclin B/Cdk1 still follows one large-amplitude peak in cyclin E/Cdk2, but the successive peaks for each variable differ by groups of three in shape and amplitude, and also by the interval between successive peaks (figure 2*c*). In the case considered in figure 2*c*, the pattern of complex periodic oscillations repeats every 96 h, i.e. four times the period of the circadian clock.

Birhythmicity reported in figure 2 is a robust phenomenon, given that similar results are obtained upon coupling the cell cycle to the circadian clock via cyclin E instead of Wee1 (see electronic supplementary material, §3, and figure S1). Birhythmicity is also generated upon coupling the cell cycle to the circadian clock through regulation by BMAL1 of both Wee1 and cyclin E. Here again we observed two domains of cell cycle entrainment to 24 h or 48 h (figure 3*a*), as previously reported [32]. In this case, as in electronic supplementary material, figure S1a, we observe two domains of birhythmicity. First, as in the case of figure 2*a* (coupling through Wee1 only) and electronic supplementary material, figure S1a (coupling through cyclin E only), a region of birhythmicity can be observed near the upper left boundary of the domain of entrainment to 48 h. This domain is larger than that delineated in figure 2*a*. Two stable oscillatory regimes coexist in this region, which, in the case considered in figure 3, have the following characteristics: one corresponds to simple periodic oscillations with a period of 48 h (figure 3*e*), while the second corresponds to complex oscillations in a pattern that repeats every 72 h (figure 3*f*). In the other instance of birhythmic behaviour illustrated in electronic supplementary material, figure S1*b*–*e*, the two modes of oscillations in each of the two domains of birhythmicity display different characteristics, as described in the legend to this figure in the electronic supplementary material.

In contrast to coupling through Wee1 only (figure 2*a*), we observed in the case of dual coupling through Wee1 and cyclin E a second, larger region of birhythmicity in the upper part of the domain of entrainment to 24 h (figure 3*a*). The phenomenon of birhythmicity in this case takes the form of a coexistence between simple periodic oscillations entrained to 24 h (figure 3*b*) and simple periodic oscillations in which sizeable oscillations in cyclin E/Cdk2 fail to be accompanied by peaks in cyclin B/Cdk1 (figure 3*c*). The latter phenomenon, referred to as endoreplication, corresponds to multiple rounds of DNA replication in the absence of mitosis. Such a phenomenon was previously reported to occur in an appropriate range of parameter values in the model of the mammalian cell cycle, in the absence of coupling to the circadian clock [33]. Endoreplication is frequently observed in plants and insects [41]. The two coexisting limit cycles shown as oscillatory time series in figure 3*b*,*c* are shown in figure 3*d* as well separated three-dimensional trajectories in phase space, projected as a function of two cell cycle variables, cyclin B/Cdk1 and cyclin E/Cdk2, and one circadian variable, nuclear BMAL1. The projections of the trajectories corresponding to the coexisting two limit cycles in figure 3*e*,*f* are very close to each other and therefore more difficult to disentangle visually.

## 4. Trirhythmicity: coexistence of three periodic attractors

In the case of dual coupling through Wee1 and cyclin E, besides the two domains of birhythmicity we observed a small region of trirhythmicity in the upper part of the domain of entrainment to 24 h, just above the domain of birhythmicity (figure 4*a*). Depending on the initial conditions, the coupled system then evolves to any one of three distinct periodic regimes: simple periodic oscillations entrained to 24 h (figure 4*b*), complex periodic oscillations entrained to 48 h (figure 4*c*) or endoreplication (figure 4*d*).

**Figure 5.**
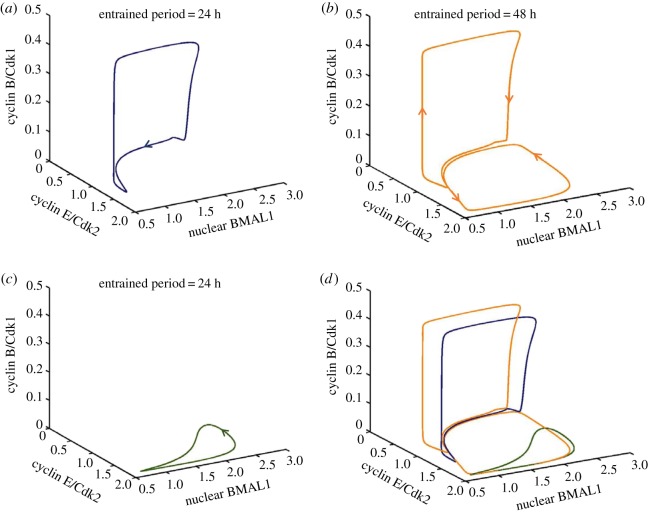
Trirhythmicity: three-dimensional projections of three coexisting limit cycles. The periodic trajectories followed in phase space by the cell cycle coupled to the circadian clock are projected as a function of cyclin B/Cdk1, cyclin E/Cdk2 and nuclear BMAL1. (*a*) Limit cycle corresponding to oscillations in figure 4*b*. (*b*) Limit cycle corresponding to complex oscillations in figure 4*c*. (*c*) Limit cycle corresponding to oscillations in figure 4*d*. (*d*) The three coexisting limit cycles shown together. (Online version in colour.)

To see how the three coexisting periodic trajectories relate to each other, it is useful to plot them in the phase space. We first show their three-dimensional projections separately, one limit cycle at a time (figure 5*a–c*), and then together (figure 5*d*), so as to better see how the different periodic trajectories are positioned in the phase space. This shows that the complex limit cycle in figure 5*b*, which corresponds to the oscillations in figure 4*c*, combines elements of the two other limit cycles in figure 5*a,c*, which correspond to the simple periodic oscillations in figure 4*b,d*.

The three oscillatory regimes encountered in the domain of trirhythmicity thus recapitulate the different types of oscillations observed in the various domains of birhythmicity, from simple oscillations entrained by the circadian clock to complex oscillations (here in the form of multiple peaks of one variable to a single peak for another variable in the cell cycle), and finally to endoreplication (oscillations in Cdk2 without oscillations of significant amplitude in Cdk1). Trirhythmicity was also found in the case where the cell cycle is entrained by the circadian clock only via cyclin E (see electronic supplementary material, §3, and figure S2).

## 5. Switching between multiple periodic attractors

In the domain of bi- or trirhythmicity, it is possible to switch from one to another periodic attractor upon suprathreshold perturbation of one or more variables. Such a switching was previously shown to occur in two- and three-variable models for birhythmicity [18,19,22,28]. Inducing the switch between two stable limit cycle trajectories proves more difficult in the coupled cell cycle–circadian clock system if only because we cannot rely on a simple phase plane representation of the trajectories to infer the phase and magnitude of the perturbations capable of inducing the transitions. Using numerical simulations, we may nevertheless search for conditions in which the transient change in one or more of the variables or parameters triggers the switch between coexisting periodic regimes. In the three cases considered, i.e. when the cell cycle is coupled to the circadian clock via Wee1 only, via cyclin E only, or via both Wee1 and cyclin E, we obtained conditions for switching and found that the transitions between multiple oscillatory regimes depend on the magnitude and duration of the perturbations, and on the phase at which the system is perturbed.

**Figure 6.**
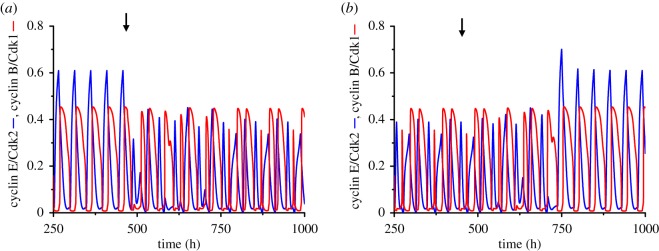
Birhythmicity: switching between two stable periodic attractors. The cell cycle is coupled to the circadian clock via Wee1 in the conditions of figure 2*b,c*. (*a*) Simple oscillations can switch to complex oscillations when the basal synthesis rate of cyclin B (*v*_cb_; in μMh^−1^) decreases from 0.055 to 0.005 for 1 h, starting at *t* = 470 h (vertical arrow). (*b*) Complex oscillations can also switch back to simple oscillations when *v*_cb_ decreases from 0.055 to 0.005 for 1 h at *t* = 453 h. The autonomous period of the cell cycle is 33.06 h (*eps* = 13) and the coupling strength *v*_sw_ is equal to 0.0146 μMh^−1^. Initial conditions are listed in electronic supplementary material, §6. (Online version in colour.)

In the case of birhythmicity, the transition from the 48 h oscillations (periodic behaviour 1, or P1, shown in figure 2*b*) to the 96 h complex oscillations (periodic behaviour 2, or P2, shown in figure 2*c*) is induced by a transient decrease in the basal rate of cyclin B synthesis, *v*_cb_ (figure 6*a*); the perturbation lasts for 1 h and starts at a phase (indicated by a vertical arrow) corresponding to time *t* = 470 h after the system starts from the initial conditions specified, as for the following figures, in electronic supplementary material, §6. Starting from P2, the reverse switch to P1 can be triggered by decreasing *v*_cb_ for 1 h when applying this transient perturbation at a phase corresponding to time *t* = 453 h (figure 6*b*). Note that induction of the switch can be rapid, as in figure 6*a*, or relatively slow and delayed, as in figure 6*b*.

**Figure 7.**
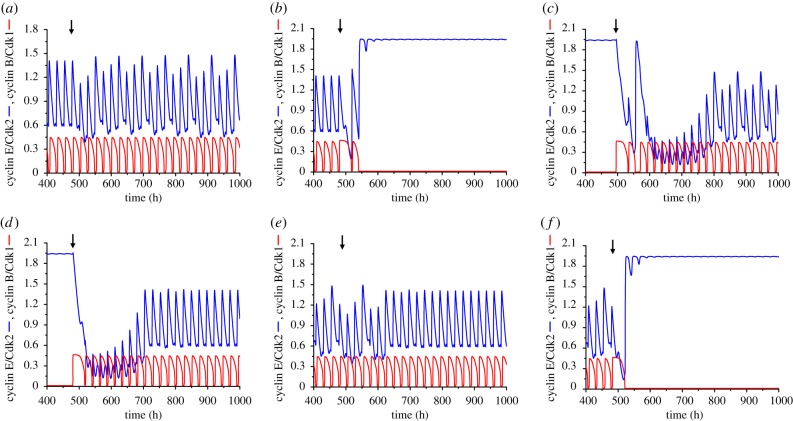
Trirhythmicity: switching between three stable periodic attractors. Conditions are those shown in electronic supplementary material, figure S2b–d, where the cell cycle is coupled to the circadian clock via cyclin E only. (*a*) Switch from simple oscillations to complex oscillations when the basal synthesis rate of cyclin B (*v*_cb_; in μMh^−1^) decreases transiently from 0.055 to 0.005, starting at *t* = 479 h (arrow); as in all panels in this figure, the transient change in *v*_cb_ lasts for 1 h. (*b*) Alternative switch from simple oscillations to small-amplitude oscillations when *v*_cb_ increases transiently from 0.055 to 0.5 at *t* = 480 h. (*c*) Switch from small-amplitude oscillations to complex oscillations triggered by a transient increase in *v*_cb_ from 0.055 to 0.5 at *t* = 495 h. (*d*) The same transient increase in *v*_cb_ from 0.055 to 0.5 starting at *t* = 480 h causes a switch from small-amplitude oscillations to simple 24 h entrainment. (*e*) Complex oscillations can switch to 24 h entrainment when *v*_cb_ decreases transiently from 0.055 to 0.005 at *t* = 480 h. (*f*) Complex oscillations can alternatively switch to small-amplitude oscillations when *v*_cb_ increases from 0.055 to 0.5 at *t* = 483 h. The autonomous period of the cell cycle is 19.36 h (*eps* = 22.2) and the coupling strength *μ*=0.06. (Online version in colour.)

In the case of trirhythmicity illustrated in electronic supplementary material, figure S2, let us denote the three coexisting oscillatory regimes by P1 (large-amplitude oscillations with a period of 24 h in electronic supplementary material, figure S2b), P2 (complex oscillations with a period of 72 h in electronic supplementary material, figure S2c) and P3 (small-amplitude oscillations with a period of 24 h in electronic supplementary material, figure S2d). Perturbations in the form of increases or decreases in the basal rate of cyclin B synthesis, *v*_cb_, lasting for 1 h and applied at different phases of the corresponding periodic solution, as detailed in the legend to figure 7, can induce the switch from P1 to P2 (figure 7*a*), P1 to P3 (figure 7*b*, note the change in scale when compared with electronic supplementary material, figure S2d), P3 to P2 (figure 7*c*), P3 to P1 (figure 7*d*), P2 to P1 (figure 7*e*) and P2 to P3 (figure 7*f*). The vertical arrows indicate the time at which the perturbation starts in each panel. Transitions between the three periodic solutions shown in figure 4*b–d* can likewise be elicited by transient changes in parameter *v*_cb_ lasting for 1 h, when applied at an appropriate phase (see electronic supplementary material, figure S3).

The same transient perturbation may trigger the switch from P3 to P2 (figure 7*c*) or to P1 (figure 7*d*), depending on the phase of P3 oscillations at which the perturbation is applied. Much as for the switch described in a two-variable system [19], and for successive switches between three stable periodic trajectories in a three-variable model for two autocatalytic enzyme reactions coupled in series [28], the transition between two periodic regimes in the coupled cell cycle–circadian clock system only occurs if the transient perturbation is applied at the right phase and with the appropriate magnitude. However, the attraction basins of the stable periodic trajectories are likely to be folded owing to the high dimensionality of the coupled cell cycle–circadian clock system. As a result, the situation is more complex than in the case of birhythmicity in a two-variable system, where the unstable periodic trajectory that separates the two stable limit cycles serves as a separatrix [19]. Here, upon perturbing one periodic solution at a given phase of one of the stable periodic regimes, we did not observe a single threshold for the duration or magnitude of the transient perturbation above which the system would quit the periodic solution to evolve to another periodic regime.

The switching between periodic regimes in the region of trirhythmicity is further illustrated in electronic supplementary material, figure S3, in the case where the cell cycle is coupled to the circadian clock via both Wee1 and cyclin E, in the conditions of figure 4*b–d*. We also show in the electronic supplementary material how the switch between periodic regimes depends on the duration (electronic supplementary material, figure S4) and magnitude (electronic supplementary material, figure S5) of the transient perturbation. Transient changes in parameters other than *v*_cb_ can also elicit the switching between multiple periodic regimes as shown in electronic supplementary material, figure S6, in the case of trirhythmicity.

The above results indicate that different types of switching can be induced by a transient increase or decrease in a parameter, depending on the phase at which the perturbation is applied and on the duration and magnitude of the perturbation. This result suggests that the structure of the attraction basins is intricate, as a result of the high dimensionality of the system. To probe the complexity of the basins' structure in view of assessing their relative sizes in the phase space so as to gain insight into which periodic solution might be the most likely in the case of bi- or trirhythmicity, we studied the evolution to one or the other periodic regime upon continuously varying the initial value of one of the variables, cyclin B/Cdk1, while starting from the same set of initial conditions for all other variables.

**Figure 8.**
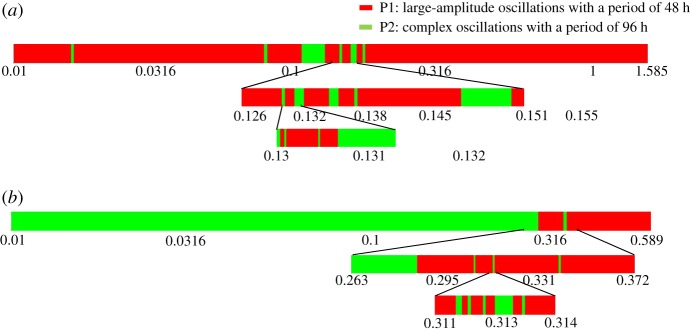
Final state sensitivity in the case of birhythmicity. (*a*) Starting from the same set of initial conditions as in figure 2*b* (large-amplitude oscillations with a period of 48 h), we progressively changed the initial value of cyclin B/Cdk1 at *t* = 470 h from 0.01 μM to 1.585 μM. (*b*) Starting from the same set of initial conditions as in figure 2*c* (complex oscillations with a period of 96 h), the value of cyclin B/Cdk1 is changed at *t* = 453 h from 0.01 μM to 0.589 μM. The red domain represents the values of cyclin B/Cdk1 leading to complex oscillations with a period of 96 h and the green domain represents the values of cyclin B/Cdk1 that lead to simple oscillations with a period of 48 h. (Online version in colour.)

To establish the effect of the initial conditions in the case of birhythmicity, we first start, as shown in figure 8*a*, from the initial conditions, as shown in figure 2*b*, corresponding to large-amplitude oscillations of 48 h, and only change the initial value of cyclin B/Cdk1 (in μM) at *t* = 470 from 0.01 to 1.585. The red domain represents the values of cyclin B/Cdk1 leading to complex oscillations with a period of 96 h and the green domain represents the values of cyclin B/Cdk1 that lead to simple oscillations with a period of 48 h. Successive enlargements confirm the complex structure of the basins of attraction. A different picture emerges in figure 8*b* if we start from the initial conditions leading in figure 2*c* to complex oscillations with a period of 96 h, and if we progressively increase the initial value of cyclin B/Cdk1 concentration at *t* = 453 h from 0.01 μM to 0.589 μM. A comparison of figure 8*a* and figure 8*b* shows that in both cases the system tends to evolve to the periodic regime from which it was initially closest. Some initial values of cyclin B/Cdk1 can nevertheless trigger the evolution to the other periodic regime. If an interval of initial values of cyclin B/Cdk1 is enlarged at finer and finer scales, we again observe a succession of initial values of cyclin B/Cdk1 leading alternatively to one of the other periodic solutions.

**Figure 9.**
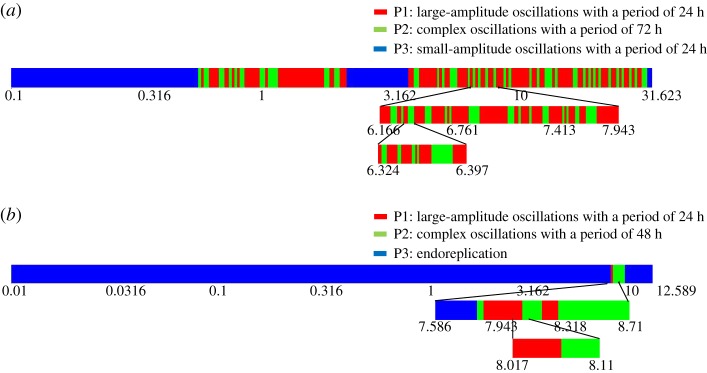
Sensitivity to initial conditions in two cases of trirhythmicity. (*a*) Starting from the same initial condition as in figure 7*d* (small-amplitude oscillations with a period of 24 h), we progressively change the value of cyclin B/Cdk1 at *t* = 480 h from 0.1 μM to 31.6228 μM. The green domain represents the initial values of cyclin B/Cdk1 leading to complex oscillations of 72 h (P2) and the red domain represents the initial values of cyclin B/Cdk1 that lead to simple oscillations with a period of 24 h (P1). The blue domain indicates the initial values of cyclin B/Cdk1 that maintain small-amplitude oscillations (P3). (*b*) Starting from the initial conditions in figure 4*d* (endoreplication), we only change the value of cyclin B/Cdk1 by progressively increasing it at *t* = 478 h from 0.1 μM to 12.5893 μM. The green domain represents the initial values of cyclin B/Cdk1 leading to complex oscillations with a period of 48 h (P2) and the red domain represents the initial values of cyclin B/Cdk1 that lead to simple oscillation with a period of 24 h (P1). The blue domain indicates the initial values of cyclin B/Cdk1 that still cause endoreplication (P3). (Online version in colour.)

Similar results are obtained in two distinct cases of trirhythmicity, as illustrated in figure 9*a,b*. The complex structure of the one-dimensional cut through the attraction basins in the cases of birhythmicity (figure 8) and trirhythmicity (figure 9) suggests that these basins are multiply folded and thus possess an intricate structure. This result is reminiscent of the situation encountered in a three-variable model displaying bi- and trirhythmicity [18,28], in which the attraction basins of the periodic regimes sometimes possess a fractal structure [42] when two stable limit cycles are separated by an unstable strange attractor.

## 6. Discussion

The coexistence of multiple oscillatory regimes, or multirhythmicity, is the rhythmic counterpart of multi-stability, which denotes the coexistence of multiple stable steady states. In its most common manifestation, multi-rhythmicity takes the form of birhythmicity in which two stable rhythms coexist. While experimental and, above all, theoretical examples of bistability abound in biological systems (see Introduction and [4]), birhythmicity appears to be less common. In a biological context, the coexistence between two modes of oscillations has been observed experimentally only in a few neuronal examples [30,31], and in a variety of models for cellular rhythms [19–26].

The abundance of biological rhythms [2,4,22,43,44] and the fact that a number of theoretical models predict multirhythmicity [18–26] raise the possibility of observing experimentally the coexistence of multiple periodic regimes in oscillating biological systems. In most models studied previously, birhythmicity arises from the interplay between two endogenous oscillatory mechanisms or between two parallel branches involving the same destabilizing feedback loop. Here, birhythmicity and trirhythmicity were found to occur in a model for the coupling of two major cellular rhythms.

Using detailed models for the cell cycle and the circadian clock in mammalian cells, we previously showed how the coupling to the circadian clock, characterized by a period of 24 h, is capable of inducing the cell cycle to oscillate at a period of 24 h or 48 h [32]. Here, we explored in further detail the dynamics of the coupled system and found evidence for multi-rhythmicity. We considered three different situations for linking the cell cycle to the circadian clock: coupling via Wee1 alone, via cyclin E alone, or via both Wee1 and cyclin E. In all these cases, we found evidence for birhythmicity, and in the cases of coupling via both Wee1 and cyclin E or only via cyclin E, we also found evidence for trirhythmicity. This is but the second example of trirhythmicity found in a system of biological interest, the first being the occurrence of three distinct oscillations that coexist in a three-variable model for two product-activated enzymes coupled in series [22,28]. What distinguishes the present findings on multi-rhythmicity from the previous reports of the phenomenon is the fact that bi- and trirhythmicity occur in a detailed, realistic model for the coupling of two major cellular rhythms, containing a much larger number of variables. Multi-rhythmicity can thus be observed in systems governed by a large number of nonlinear, coupled differential equations, much as in simpler models containing but a few variables.

In some of the situations considered above, one of the periodic solutions has only a minute amplitude. This occurs for the cases of birhythmicity (electronic supplementary material, figures S1c and S1e) and trithyhmicity (electronic supplementary material, figure S2d). From a physiological point of view, such a small-amplitude periodic solution might be perceived by the cell as a steady state, which would then coexist with either one or two limit cycles corresponding to large-amplitude oscillations. The small-amplitude oscillations would probably be associated with cell cycle arrest in the S phase, because the level of cyclin E/Cdk2 (controlling the DNA replication) is relatively high, while the level of cyclin B/Cdk1 (controlling the M phase) is low.

In regard to switching between multiple periodic regimes, the results indicate that the transition between two stable rhythms only occurs if the transient perturbation is applied at the right phase, with the appropriate duration and magnitude. This result is reminiscent of those obtained for the suppression of circadian rhythmicity by a pulse of light in a model for the *Drosophila* circadian clock in conditions of hard excitation [17].

Because of the large dimensionality of the coupled cell cycle–circadian clock system, the structure of the attraction basins in the case of multi-rhythmicity appears to be complex, as suggested by the finding that, at a given phase of the oscillations, the progressive modification of the duration or magnitude of the transient change of a control parameter can alternatively lead to different periodic solutions. A similar final state sensitivity was observed in the cases of birhythmicity (figure 8) and two different instances of trirhythmicity (figure 9) upon progressively changing the initial value of a key variable, cyclin B/Cdk1, while starting from the same set of initial conditions for the remaining variables. Such sensitivity likely results from the multiple folding of trajectories due to the high dimensionality of the differential system, which contains some 60 variables. This phenomenon is reminiscent of the situation in which fractal basin boundaries were found in a three-variable system displaying birhythmicity, although in the latter case the phenomenon occurred in conditions where the two stable limit cycles appeared to be separated by an unstable strange attractor associated with chaos [42]. It could also be related to intermingled [45] or rugged attraction basins [46,47] observed for multiple attractors in physical systems. The phenomenon of final state sensitivity is of physical and biological significance as it might represent an obstruction to predictability [45–48].

Because of the complex structure of the attraction basins, it is difficult to draw predictions about which is the most likely periodic solution that will be established in the case of multi-rhythmicity. The results in figures 8 and 9 indicate, moreover, that, even though a similar intermingled structure is found for the one-dimensional cut through the attraction basins, the system tends to evolve preferentially towards the periodic solution from which it was initially closest in the phase space. The results in figures 8 and 9 nevertheless suggest that, in conditions of multi-rhythmicity, switches between different periodic attractors might occur spontaneously owing to fluctuations in the levels of some key cell cycle proteins.

The occurrence of multi-rhythmicity was demonstrated here in a high-dimensional system based on the coupling of detailed models for the cell cycle and the circadian clock. That multi-rhythmicity occurs in a realistic model for the coupling of two major cellular rhythms raises the likelihood of observing the phenomenon in biological systems. But, the final state sensitivity revealed by simulations of the effect of initial conditions or of transient perturbations in the situations of bi- or trirhythmicity strengthens interest in considering the dynamics of a system of two coupled cellular rhythms of high dimensionality.

Even if multi-rhythmicity occurs here in the absence of external periodic forcing, we may view the coupling of the cell cycle to the circadian clock as a form of internal forcing of the former oscillator by a circadian rhythm. This situation is reminiscent of the three-variable biochemical model in which birhythmicity and trirhythmicity were initially found [18,22,28]. The interest of the latter model is to allow a detailed bifurcation analysis, which is not possible in a system of some 60 variables as that considered here. Schematized in electronic supplementary material, figure S7, the bifurcation diagram obtained for the three-variable model [28] provides a scenario for the onset of bi- and trirhythmicity. It might be difficult to test in the fully detailed model if this scenario, established in a three-variable model, is generic for bi- and trirhythmicity. An alternative approach may consist in using simplified versions of the models for the circadian clock and the cell cycle. Thus a skeleton model of five variables (instead of 41 variables for the fully detailed model) was previously proposed for the mammalian cell cycle [25], while models containing five or even three variables were studied for the *Drosophila* and *Neurospora* circadian clocks [49,50]. The latter models could be amended to represent the oscillatory behaviour of the mammalian circadian clock. Coupling the reduced models for the cell cycle and the circadian clock would allow us to search for multi-rhythmicity in a system containing 10 variables or fewer, which would be more amenable to numerical and bifurcation analysis. If the occurrence of multi-rhythmicity were to be confirmed in such a reduced system, it would be interesting to determine whether the phenomenon remains characterized by the final state sensitivity found in the detailed, more realistic model considered here.

Most experimental observations on the links between the cell cycle and circadian rhythms point to the circadian control of a number of cell cycle proteins, and thereby support the view of a unidirectional coupling of the cell cycle to the circadian clock. Several studies nevertheless suggest that the cell cycle may, in turn, impinge on the dynamics of the circadian clock [51,52]. We recently examined the consequences of such bidirectional coupling and also found evidence for bi- and trirhythmicity in these conditions [53]. This finding corroborates the results of the present study on the occurrence of multi-rhythmicity generated by the coupling of two cellular rhythms. In the presence of bidirectional coupling, instead of a coexistence between different modes of entrained oscillations, multi-rhythmicity takes the form of a coexistence between two or three modes of synchronization at periods that differ from the autonomous periods of the cell cycle and circadian clock; moreover, in each mode of synchronization, the oscillations possess a simple periodic form.

The question arises as to the physiological significance of multi-rhythmicity. In this respect, it is useful to compare rhythmic behaviour with that of a system operating in a stable steady state, and start by comparing monorhythmicity with monostability. Biological rhythms of the limit cycle type play many key roles in physiology and in the origin of a large number of disorders (see [44] for a recent review). Monostability, i.e. the evolution to a unique steady state, is more common, and perhaps less remarkable from a dynamical point of view, even if the maintenance of a steady state in an appropriate range is of paramount importance in physiology, as stressed by Claude Bernard [54], who developed the concept of constancy of the ‘milieu intérieur’, and by Cannon [55], for whom a stable steady state needs to be maintained through feedback mechanisms ensuring homeostasis. If multi-stability breaks homeostasis, it nevertheless introduces the capability of switching between multiple steady states. This enrichment in the repertoire of non-equilibrium dynamical behaviour provides new biological mechanisms for memory, switch-like responses and cell differentiation [2,4,10–15].

The roles of bistability have been discussed in regard to a variety of diseases. Thus, in models accounting for bistable transitions, one branch of stable steady states often corresponds to the healthy state, while the other branch of stable steady states is associated with a particular disease (see [4] for a recent review). The field where multiple steady states are thought to be frequently encountered is definitely that of developmental biology. Bistability and tristability indeed appear to play a primary role in the determination of cell fate [12–15] in the course of cell differentiation.

What about the possible roles of bi- or trirhythmicity? The coexistence between several oscillatory states might play positive roles, which remain to be explored. In a cell population, in conditions of multi-rhythmicity, because of intracellular fluctuations a distribution of cells between distinct periodic attractors may be expected, much as in the case of a coexistence between a stable limit cycle and a stable steady state in a skeleton model for the mammalian cell cycle [56]. Multi-rhythmicity would then provide a source of heterogeneity within a cell population. Some cells might be endowed with a proliferation advantage, depending on the particular periodic attractor towards which their cell cycle evolves. Some of these attractors may be associated with endoreplication (figure 4*d*) or with two rounds of DNA replication in each division cycle (figure 4*b*). The phenotype associated with more complex oscillatory behaviour (figure 6*a*) remains unclear, and such oscillations might perhaps be lethal for the cell. Interestingly, complex modes of oscillations tend to be replaced by simple periodic oscillations when coupling between the cell cycle and the circadian clock becomes bidirectional [53].

Before addressing the physiological significance of multirhythmicity, however, the occurrence of the phenomenon needs to be established experimentally. By clarifying the conditions in which it occurs, theoretical models such as the one considered here will hopefully contribute to motivate and guide the investigation of birhythmicity and trirhythmicity in biological systems. One physiological system of particular importance which could be investigated in this light is the cardiorespiratory system. The cardiac and the respiratory rhythms interact and can synchronize over sizeable lapses of time; spontaneous switches between different patterns of synchronization can be observed [57]. Moreover, the pattern of cardiorespiratory synchronization changes as a function of sleep stage and age [58]. It is not yet clear whether multiple modes of synchronization between the coupled cardiac and respiratory rhythms coexist in a given set of conditions, but the present results obtained for multi-rhythmicity in a cellular context suggest such a possibility.

## Supplementary Material

Supporting Information

## Data accessibility

The computer code used for numerical simulations and all parameter values and initial conditions needed to reproduce the results are listed in the electronic supplementary material.
