# Supplementary material for: Multi-rhythmicity generated by coupling two cellular rhythms
Source: J R Soc Interface. 2019 Mar 6;16(152):20180835. doi: 10.1098/rsif.2018.0835 (PMC6451392; doi:10.1098/rsif.2018.0835)
Supplement: Supporting Information [file rsif20180835supp1.docx]

**“Multirhythmicity generated by coupling two cellular rhythms”**

Jie Yan and Albert Goldbeter

**Supporting information**

**1. Model for the coupling of the cell cycle to the circadian clock** ……………….…….. 2

**2. Three ways of coupling the cell cycle to the circadian clock** ……………………….. 2

**3. Birhythmicity and trirhythmicity when the cell cycle is coupled to the
 circadian clock via cyclin E** ………**……………………………….…………………..** 3

**4. Switching between multiple periodic regimes and final state sensitivity** ………….. 4

**5. A bifurcation scenario for multirhythmicity** ………………….…………………….. 5

**6. Initial conditions for observing multiple periodic attractors** ……………...……….. 6

**7. Figures S1 to S7** ……………..…………………….……………..……………....……. 10

**8. References** ……………………………………….…………………………...………… 18

**9. Computer code used in numerical simulations of the model for the
 coupling of the cell cycle to the circadian clock** …………………….……...….…….. 19

**1. Model for the coupling of the cell cycle to the circadian clock**

In this study, to investigate the possibility of a coexistence between multiple periodic attractors we used a detailed mathematical model describing the dynamics of the Cdk network driving the mammalian cell cycle [1] and a mathematical model proposed for the mammalian circadian clock, in the version that includes the regulation of Bmal1 by the protein Rev-Erbα [2,3]. With the exception of a few minor modifications described in Section 2 below we followed the approach developed in a previous study of entrainment of the cell cycle by the circadian clock [4].

The kinetic equations governing the time evolution of the model for the Cdk network are listed as eqs. [1]–[39] in Supporting Information of ref. [1]; see:

<http://www.pnas.org/content/106/51/21643/tab-figures-data>

The definition and values of the parameters of the cell cycle model are listed at the same address, in Table S2, with *v_cb_*=0.055μM h-^1^.

The kinetic equations describing the time evolution of the model for the mammalian circadian clock are listed in the Supporting Information of ref. [2]. See:

<http://www.pnas.org/content/100/12/7051/tab-figures-data>

Parameter values for the circadian clock model are listed at the same address in the legend to Supporting Figure 8, with *K*_ib_ =1 nM. See:

**2.** **Three ways of coupling the cell cycle to the circadian clock**

Experiments have shown that some of the components of cell cycle, such as Wee1, cyclin E and p21, are regulated by the circadian clock. In this manuscript, following our previous approach [4], we considered three different ways of coupling the cell cycle to the circadian clock. In each situation, we explored the conditions in which multiple periodic attractors occur.

We first considered the coupling of the cell cycle to the circadian clock via Wee1, a protein kinase that inhibits cyclin-dependent kinases such as Cdk1 [5]. Previous experimental data indicated that the circadian clock can regulate the cell cycle by promoting the transcription of *wee1* mRNA through the BMAL1/CLOCK complexes [6]. Therefore, we incorporated an additional kinetic equation for the *Wee1* mRNA, to describe this coupling of the cell cycle to the circadian clock. The time evolution of the concentrations of Wee1 mRNA (*Mw*) and Wee1 protein (*Wee1*) is thus governed by eqs. (1) and (2) below, where the rate of synthesis of *Wee1* mRNA dependent on BMAL1 (*B*_n_), denoted *v_sw_*, measures the coupling strength of the cell cycle to the circadian clock:

$\frac{dMw}{dt}=v_{swee1}+v_{sw}\cdot\frac{{B_{n}}^{nmw}}{{K_{aw}}^{nmw}+{B_{n}}^{nmw}}-V_{dmw}\cdot\frac{Mw}{K_{dmw}+Mw}$ (1)

$\frac{dWee1}{dt}={(k}_{sw}\cdot Mw-V_{m7b}\cdot\left( Mb+i_{b} \right)\cdot\frac{Wee1}{K_{7b}+Wee1}+V_{m8b}\cdot\frac{{Wee1}_{p}}{K_{8b}+{Wee1}_{p}}-k_{dwee1}\cdot Wee1)\cdot eps$ (2)

The coupling term via Wee1 was slightly modified with respect to our previous study of coupling the cell cycle to the circadian clock, i.e. with respect to eqs. (1) and (2) in ref. [4]. Here, we assumed that *v*_swee1_ is the basal rate of *Wee1* mRNA synthesis, rather than Wee1 protein. This is reasonable because the basal synthesis of Wee1 protein, which is independent of circadian clock, also depends on *Wee1* mRNA. Parameter values in eqs. (1) and (2) above are: *v_swee1_* =0.0117 μM h^-1^, *nmw*=4, *K_aw_* =2 nM, *V_dmw_*=0.5 μM h^-1^, *K_dmw_* =0.5 μM.

The second way considered for coupling the cell cycle to the circadian clock is via cyclin E [4], since the BMAL1/CLOCK complex appears to repress the expression of cyclin E gene indirectly [7,8]. Therefore, we added a kinetic equation for cyclin E mRNA in the model describing the coupling of the cell cycle to the circadian clock. The time evolution of the concentrations of cyclin E mRNA (*M*_ce_) and cyclin E protein (*C*_e_) is governed by eqs. (3) and (4) below, where the rate of synthesis of cyclin E mRNA inhibited by BMAL1, *v_sce_*, measures the coupling strength of the cell cycle to the circadian clock:

$\frac{dMCe}{dt}=v_{sce}\cdot\frac{{K_{ice}}^{nce}}{{K_{ice}}^{nce}+{B_{n}}^{nce}}-V_{dmce}\cdot\frac{Mce}{K_{dmce}+Mce}$ (3)

$\frac{dCe}{dt}={(k}_{ce}\cdot E2F\cdot\frac{K_{i9}}{K_{i9}+pRB}\cdot\frac{K_{i10}}{K_{i10}+pRB_{p}}+k_{ce2}\cdot Mce-k_{com2}\cdot Ce\cdot\left( Cdk2_{tot}-\left( Mei+Me+Mep27+Mai+Ma+Map27 \right) \right)+k_{decom2}\cdot Mei-V_{de}\cdot\frac{Skp2}{K_{dceskp2}+Skp2}\cdot\frac{Ce}{K_{de}+Ce}-k_{dde}\cdot Ce)\cdot eps$

(4)

Equations (3) and (4) are the same as eqs. (5) and (6) considered by Gérard and Goldbeter [4] for the coupling of the cell cycle to the circadian clock via cyclin E. Parameter values in eqs. (3) and (4) are *K_ice_*=1nM, *V_dmce_* =0.5μM h^-1^, *K_dmce_* =0.5 μM, *nce*=4, *k_ce2_* =5 h^-1^.

Finally we considered a third mode of coupling via both Wee1 and cyclin E. We multiply *v_sw_* and *v_sce_* by the same dimensionless parameter *μ*. The values of *v_sw_* and *v_sce_* are equal to 1 nM h^-1^. The multiplicative factor *μ* measures the strength of coupling.

**3. Birhythmicity and trirhythmicity when the cell cycle is coupled to the circadian clock via cyclin E**

Birhythmicity shown in Fig. 2 is a robust phenomenon, given that similar results are obtained upon coupling the cell cycle to the circadian clock via cyclin E instead of Wee1. In this case a region of birhythmicity is found in a large area in the upper part of the domains of entrainment to 24h or 48h (see Fig. S1). The coexistence of two periodic regimes is illustrated in each of the two domains of birhythmicity: see Figs. S1(b)-(c) and S1(d)-(e). In both cases, one of the stable periodic regimes corresponds to large-amplitude oscillations entrained at 24h [Fig. S1(b)] or 48h [Fig. S1(d)], while the other periodic regime corresponds to small-amplitude oscillations entrained to 24h [Figs. S1(c)] and S1(e)].

Trirhythmicity was also found in the case where the cell cycle is entrained by the circadian clock only via cyclin E (see Fig. S2). In these conditions we saw in Fig. S1(a) that birhythmicity occurs above the red and blue boundaries in the upper parts of the domains of entrainment to 24h and 48h, respectively. Trirhythmicity occurs in a narrow area located to the left boundary of the region of birhythmicity, in the domain of entrainment to 24h in Fig. S2(a). In this region of trirhythmicity, depending on initial conditions, the cell cycle coupled to the circadian clock can evolve to either one of the following three periodic modes of oscillations which, in the case considered in Fig. S2, have the following characteristics: simple periodic oscillations of 24h period (b), complex oscillations of 72h period (i.e. 3 times the forcing period) in which three nearly identical peaks in Cdk1, spaced by nearly equal intervals close to 24h, are accompanied by three peaks of different shape and amplitude in cyclin E/Cdk2 per period (c), and small-amplitude oscillations (d).

**4. Switching between multiple periodic regimes and final state sensitivity**

In the case of a two-variable model in which birhythmicity was previously studied (see ref. [19] of main text), it was easy to plot the two stable limit cycles in the phase plane, and to determine, by selecting suitable initial conditions, the unstable trajectory that separates these two stable cycles. The switch from the low-amplitude limit cycle to the surrounding large-amplitude limit cycle can be achieved when increasing instantaneously above a threshold corresponding to the unstable periodic trajectory, the concentration of the variable that is near its minimum [19]. The reverse transition towards the small-amplitude limit cycle can similarly be triggered by a suprathreshold decrease in this variable when it is close to its maximum.

We showed in Figs. 6 and 7 in the main text how a transient change in a parameter can trigger the switch between alternative stable periodic regimes in the cases of birhythmicity and trirhythmicity, respectively. An additional example is shown in Fig. S3 for the case of trirhythmicity when the cell cycle is coupled to the circadian clock via both Wee1 and cyclin E. Switching between periodic regimes depends on the duration of the transient perturbation. This is illustrated in Fig. S4 in the case of trirhythmicity described in Figs. 7 and S2.

In Figs. 6, 7, and S3, we showed that different types of switching can be induced by a transient increase or decrease in parameter *v*_cb_ lasting for 1h, depending on the phase at which the perturbation is applied. At a given phase, the perturbation may also lead to one or another periodic solution, depending on the duration and magnitude of the perturbation. Thus, in the conditions of Fig. 7a, at t=479h, when *v*_cb_ decreases transiently from 0.055 to 0.005, simple oscillations fail to switch to another periodic attractor if the duration of the change is between 0.1h and 0.6h, 1.1h and 1.2h, 1.7h and 1.8h, or 2h. If the duration of the change is between 0.7h and 1h, 1.3 and 1.6h or 1.9h, simple oscillations will switch to complex oscillations (Fig 7a).

In the conditions of Fig. 7b, at t=480h, when *v*_cb_ increases transiently from 0.055 to 0.5, if the duration of the change is within the intervals 0.1-0.3h, 0.7-0.8h, 1.4-1.5h, or 1.7-1.9h, simple oscillations fail to switch to another periodic attractor. If the duration of the change lasts between 1h and 1.2h, the simple oscillations will switch to small-amplitude oscillations (see Fig. S4(a), where the duration of the perturbation is equal to 1h). If the duration of the change is within the interval 0.4-0.6h, or equal to 0.9h, 1.3h, 1.6 or 2h, simple oscillations will switch to complex oscillations (see Fig. S4(b) where the perturbation lasts 0.4h).

A similar sensitivity is observed with respect to the magnitude of the change in *v*_cb_, if the duration of the perturbation and the phase at which it starts are fixed. Thus, in the conditions of Fig. S3(a), at t=480h, if the magnitude of *v*_cb_ transiently changes for 1h from an initial value of 0.055 to a value within the intervals 0.026-0.064, 0.954-1.57, 1.584-1.595 or 1.598-1.599, simple oscillations fail to switch to another periodic regime. If *v*_cb_ is changed to a value within the intervals 0-0.025, 0.065-0.953, 1.571-1.583 or 1.596-1.597, simple oscillations switch to complex oscillations (see Fig. S5(a), where the parameter transiently changes to the value *v*_cb_ =0.005). If *v*_cb_ is increased to a value in the interval 1.6-2, simple oscillations switch to endoreplication (Fig. S5(b), for a transient change to the value *v*_cb_ =1.6).

The switch between multiple periodic attractors was triggered in Figs. 6 and 7, as well as in Figs. S3-S5, by a transient change in the basal rate of cyclin B synthesis, *v*_cb_. Other parameters can be used to elicit the transition between different periodic regimes in the cases of bi- or trirhythmicity. This is illustrated in Fig. S6 for the trirhythmic situation considered in Fig. S2. Starting from the initial conditions taken in Fig. S2(c), a transient decrease in the total Cdk2 concentration (Cdk2_tot_) for 1 hour triggers a switch from the complex oscillations of 72 h period to simple oscillations of 24h period (a). A similar switch is obtained in (b) by a transient increase in the basal rate of wee1 mRNA (*v_swee1_*) synthesis. Finally, starting from the initial conditions taken in Fig. S2(b), simple oscillations switch to complex oscillations in (c) following a transient decrease in the total Cdk1 concentration (Cdk1_tot_).

**5. A bifurcation scenario for multirhythmicity**

Birhythmicity and trirhythmicity were initially found in a 3-variable model for two oscillatory enzyme reactions coupled in series (see refs [18,22,28] in main text). The interest of the latter, simpler model is to allow a detailed bifurcation analysis, which is not possible in a system of some 60 variables as that considered here. Schematized in Fig. S7, the bifurcation diagram obtained for the 3-variable model, adapted from ref. [28] in main text), provides a scenario for the onset of bi- and trirhythmicity. Upon continuously increasing a control parameter, we first encounter in this model a region of sustained oscillations around an unstable steady state (denoted X_0_) represented by one of the variables, X. The oscillations correspond to a first limit cycle (LC1), which originates through a subcritical Hopf bifurcation when the stable steady state (X_0_, solid line) becomes unstable (X_0_, dashed line). Over a certain range of the control parameter the stable steady state coexists with the stable periodic solution LC1, from which it is separated by an unstable periodic trajectory. Upon further increasing the control parameter, the steady state X_0_ becomes unstable again, at a supercritical Hopf bifurcation where a second periodic solution (LC2) appears. If LC2 appears in the range where LC1 is still present, birhythmicity ensues. Moreover, if a hysteresis loop develops between limit cycle LC2 and a third limit cycle LC3 upon further increasing the control parameter, a new domain of birhythmicity is created. If this overlaps with the domain of existence of LC1, the three periodic solutions LC1, LC2 and LC3 coexist. Such a situation corresponds to trirhythmicity.

**6. Initial conditions** **for observing multiple periodic attractors**

The differential equations that govern the time evolution of the model were integrated numerically by means of the Runge-Kutta method provided in the program XPPAUTO developed by Dr. Bard Ermentrout at the University of Pittsburgh [9]. The observation of multiple periodic attractors is highly sensitive to initial conditions. Initial conditions for the figures in the text and in Supporting information are listed below (concentration units are expressed in nM for circadian variables (see Supporting information in [3]) and in μM for cell cycle variables (see Supporting information in [1]) :

Fig. 2b and Fig. 6a Mp=4.0342002, Mc=4.6187, Mbmal=8.4909, Pc=0.023964999, Cc=327.7673, Pcp=0.013851, Ccp=0.76131999, PCc=3.9844, PCn=1.0813,PCcp=5.3548002, PCnp=0.62621999, Bc=3.3185, Bcp=0.14084999, Bn=1.7053, Bnp=0.090561002, In=1.6584001, Mr=2.6716001, Rc=1.5322, Rn=1.1181999, AP1=6.0605998, pRB=1.4225, pRBc1=1.567, pRBp=12.2283, pRBc2=6.7241998, pRBpp=0.00099184003, E2F=11.1372, E2Fp=0.0077661001, Cd=0.094114996, Mdi=0.021430001, Md=0.74659997, Mdp27=0.59710002, Mce=0, Ce=0.0011064, Mei=0.0061730999, Me=0.019832, Skp2=21.161699, Mep27=0.012164, Pei=0.17065001, Pe=1.3978, Ca=0.003338, Mai=0.011651, Ma=0.0035418, Map27=0.0011525, p27=0.32516, p27p=0.018523, Cdh1i=0.54514003, Cdh1a=0.0093810996, Pai=0.59320003, Pa=0.21314, Cb=0.22935, Mbi=0.039067999, Mb=0.34164, Mbp27=0.062483002, Cdc20i=0.038474001, Cdc20a=1.8981, Pbi=0.081721999, Pb=1.0404, Mw=0.019308999, Wee1=0.20207, Wee1p=0.39875999.

Fig. 2c and Fig. 6b: Mp=3.5994, Mc=4.1381998, Mbmal=10.0127, Pc=0.021329001, Cc=318.6214, Pcp=0.012222, Ccp=0.76115, PCc=3.1317999, PCn=0.62753999,PCcp=4.2498999, PCnp=0.31399, Bc=3.9242001, Bcp=0.14819001, Bn=2.5016, Bnp=0.10739, In=0.87125999, Mr=2.4974, Rc=1.0913, Rn=0.56395, AP1=6.0605998, pRB=1.3906, pRBc1=1.5869, pRBp=12.5438, pRBc2=7.1753001, pRBpp=0.014838, E2F=11.5112, E2Fp=0.058377001, Cd=0.094130002, Mdi=0.022756999, Md=1.2779, Mdp27=0.067538001, Mce=0, Ce=0.022678999, Mei=0.054009002, Me=0.26111999, Skp2=0.11388, Mep27=0.0084520001, Pei=0.052152999, Pe=1.6274, Ca=0.11267, Mai=0.29085001, Ma=0.026218001, Map27=0.00050924998, p27=0.016170001, p27p=0.043696001, Cdh1i=0.0028945, Cdh1a=1.0943, Pai=0.55353999, Pa=0.29100999, Cb=0.11101, Mbi=0.11006, Mb=0.0092435004, Mbp27=9.2194998e-05, Cdc20i=0.71082002, Cdc20a=0.010123, Pbi=0.55111003, Pb=0.097972997, Mw=0.023360001, Wee1=0.62681001, Wee1p=0.27224001.

Fig. S1(b): Mp=4.0342, Mc=4.6187, Mbmal=8.4909, Pc=0.02396, Cc=327.7673, Pcp=0.01385, Ccp=0.76132, PCc=3.9844, PCn=1.0813, PCcp=5.3548, PCnp=0.62622, Bc=3.3185, Bcp=0.14085, Bn=1.7053, Bnp=0.09056, In=1.6584, Mr=2.6716, Rc=1.5322, Rn=1.1182 AP1=6.0606, pRB=1.4878, pRBc1=2.2087, pRBp=12.227, pRBc2=9.0762, pRBpp=0.05742, E2F=14.9737, E2Fp=0.00819, Cd=0.09413, Mdi=0.02283, Md=1.3177, Mdp27=0.02085, Mce=0.00645, Ce=0.00278, Mei=0.02898, Me=0.73755, Skp2=17.427, Mep27=0.00825, Pei=0.02175, Pe=1.6906, Ca=0.00438, Mai=0.00979, Ma=0.00343, Map27=2.1468E-5, p27=0.0056, p27p=0.01954, Cdh1i=0.54659, Cdh1a=0.00675, Pai=0.59338, Pa=0.21284, Cb=0.2077, Mbi=0.03326, Mb=0.41118, Mbp27=0.00137, Cdc20i=0.0301, Cdc20a=1.9169, Pbi=0.06449, Pb=1.0719, Mw=0.01198, Wee1=0.11797, Wee1p=0.2403.

Fig. S1(c): Mp=3.2219, Mc=4.0914, Mbmal=8.8284, Pc=0.0151, Cc=412.95889, Pcp=0.0086, Ccp=0.7633, PCc=3.0569, PCn=0.8567, PCcp=11.8592, PCnp=0.5722, Bc=3.0471, Bcp=0.1358, Bn=1.5129, Bnp=0.0841, In=0.7315, Mr=2.0935, Rc=0.7221, Rn=0.3503, AP1=0.1, pRB=0.1, pRBc1=0.1, pRBp=0.1, pRBc2=0.1, pRBpp=0.1, E2F=0.1, E2Fp=0.1, Cd=0.1, Mdi=0.1, Md=0.1, Mdp27=0.1, Mce=0.1, Ce=0.1, Mei=0.1, Me=0.1, Skp2=0.1, Mep27=0.1, Pei=0.1, Pe=0.1, Ca=0.1, Mai=0.1, Ma=0.1, Map27=0.1, p27=0.1, p27p=0.1, Cdh1i=0.1, Cdh1a=0.1, Pai=0.1, Pa=0.1, Cb=0.1, Mbi=0.1, Mb=0.1, Mbp27=0.1, Cdc20i=0.1, Cdc20a=0.1, Pbi=0.1, Pb=0.1, Mw=0.1, Wee1=0.1, Wee1p=0.1.

Fig. S1(d): Mp=4.0342, Mc=4.6187, Mbmal=8.4909, Pc=0.02396, Cc=327.7673, Pcp=0.01385, Ccp=0.76132, PCc=3.9844, PCn=1.0813, PCcp=5.3548, PCnp=0.62622, Bc=3.3185, Bcp=0.14085, Bn=1.7053, Bnp=0.09056, In=1.6584, Mr=2.6716, Rc=1.5322, Rn=1.1182, AP1=6.0606, pRB=1.4932, pRBc1=2.6417, pRBp=12.2706, pRBc2=10.854, pRBpp=0.05735, E2F=17.8103, E2Fp=0.01016, Cd=0.09414, Mdi=0.02283, Md=1.3176, Mdp27=0.02092, Mce=0.00429, Ce=0.00262, Mei=0.02917, Me=0.7369, Skp2=18.4977, Mep27=0.00827, Pei=0.02176, Pe=1.6906, Ca=0.0052, Mai=0.01174, Ma=0.00408, Map27=2.58E-5, p27=0.00562, p27p=0.0196, Cdh1i=0.54633, Cdh1a=0.00724, Pai=0.59243, Pa=0.21474, Cb=0.14998, Mbi=0.03339, Mb=1, Mbp27=0.00132, Cdc20i=0.03185, Cdc20a=1.9128, Pbi=0.06812, Pb=1.0651, Mw=0.01198, Wee1=0.12131, Wee1p=0.23855.

Fig. S1(e): Mp=3.2219, Mc=4.0914, Mbmal=8.8284, Pc=0.0151, Cc=412.95889, Pcp=0.0086, Ccp=0.7633, PCc=3.0569, PCn=0.8567, PCcp=11.8592, PCnp=0.5722, Bc=3.0471, Bcp=0.1358, Bn=1.5129, Bnp=0.0841, In=0.7315, Mr=2.0935, Rc=0.7221, Rn=0.3503, AP1=0.1, pRB=0.1, pRBc1=0.1, pRBp=0.1, pRBc2=0.1, pRBpp=0.1, E2F=0.1, E2Fp=0.1, Cd=0.1, Mdi=0.1, Md=0.1, Mdp27=0.1, Mce=0.1, Ce=0.1, Mei=0.1, Me=0.1, Skp2=0.1, Mep27=0.1, Pei=0.1, Pe=0.1, Ca=0.1, Mai=0.1, Ma=0.1, Map27=0.1, p27=0.1, p27p=0.1, Cdh1i=0.1, Cdh1a=0.1, Pai=0.1, Pa=0.1, Cb=0.1, Mbi=0.1, Mb=0.1, Mbp27=0.1, Cdc20i=0.1, Cdc20a=0.1, Pbi=0.1, Pb=0.1, Mw=0.1, Wee1=0.1, Wee1p=0.1.

Fig.3b: Mp=1.0003, Mc=1.698, Mbmal=6.805, Pc=0.0055822, Cc=328.4629, Pcp=0.0031028, Ccp=0.76158, PCc=0.75418, PCn=0.2771, PCcp=5.8789, PCnp=0.44121, Bc=1.9198, Bcp=0.1102, Bn=1.0386, Bnp=0.065823, In=0.070079, Mr=0.82908, Rc=0.12613, Rn=0.036569 AP1=6.0606, pRB=1.5921, pRBc1=0.20208, pRBp=13.5477, pRBc2=0.86082, pRBpp=0.091333, E2F=1.2949, E2Fp=0.11011, Cd=0.094086, Mdi=0.022841, Md=1.3225, Mdp27=0.016087, Mce=0.028122, Ce=0.006114, Mei=0.029935, Me=0.96505, Skp2=10.6707, Mep27=0.0081096, Pei=0.017005, Pe=1.6997, Ca=0.0044513, Mai=0.051508, Ma=0.16472, Map27=0.00099041, p27=0.0042028, p27p=0.019275, Cdh1i=0.54806, Cdh1a=0.0037106, Pai=0.20488, Pa=1.0414, Cb=1.0856, Mbi=0.033732, Mb=0.45296, Mbp27=0.0011308, Cdc20i=0.026537, Cdc20a=1.8263, Pbi=0.057179, Pb=1.0824, Mw=0.013442, Wee1=0.11901, Wee1p=0.26963.

Fig.3c: Mp=3.4509, Mc=3.984, Mbmal=10.0391, Pc=0.020401, Cc=317.3298, Pcp=0.011662, Ccp=0.76114, PCc=2.9193, PCn=0.56669, PCcp=4.0657, PCnp=0.27604, Bc=3.9105, Bcp=0.14791, Bn=2.5688, Bnp=0.10847, In=0.7199, Mr=2.4211, Rc=0.98579, Rn=0.47282, AP1=6.0606, pRB=1.576, pRBc1=10.4992, pRBp=12.81, pRBc2=42.6575, pRBpp=0.29481, E2F=66.6106, E2Fp=0.018566, Cd=0.094285, Mdi=0.022849, Md=1.3276, Mdp27=0.011106, Mce=0.00088102, Ce=8.783, Mei=0.34986, Me=1.5161, Skp2=0.11373, Mep27=0.0086715, Pei=0.011127,Pe=1.7112, Ca=2.7717, Mai=0.099727, Ma=0.006256, Map27=2.053e-05, p27=0.0028624, p27p=0.047187, Cdh1i=0.00074166, Cdh1a=1.0985, Pai=0.58913, Pa=0.22117, Cb=0.1207, Mbi=0.11404, Mb=0.0046496, Mbp27=7.936e-06, Cdc20i=0.71255, Cdc20a=0.0048493, Pbi=0.55448, Pb=0.091041, Mw=0.044751, Wee1=1.5275, Wee1p=0.35309.

Fig.3e Mp=3.2463, Mc=3.7733, Mbmal=9.9569, Pc=0.019106, Cc=316.0825, Pcp=0.010885, Ccp=0.76114, PCc=2.6433, PCn=0.49884, PCcp=3.8667, PCnp=0.23522, Bc=3.8409, Bcp=0.14693, Bn=2.6083, Bnp=0.10903, In=0.55564, Mr=2.3117, Rc=0.85671, Rn=0.37449, AP1=6.0606, pRB=1.4825, pRBc1=3.6321, pRBp=12.9241, pRBc2=15.8455, pRBpp=0.020356, E2F=24.4996, E2Fp=0.1303, Cd=0.094164, Mdi=0.022624, Md=1.1951, Mdp27=0.14775, Mce=0.00015657, Ce=0.048424, Mei=0.044671, Me=0.34226, Skp2=0.11407, Mep27=0.0087755, Pei=0.042149, Pe=1.6245, Ca=0.11943, Mai=0.26814, Ma=0.049825, Map27=0.00089792, p27=0.012494, p27p=0.044846, Cdh1i=0.0060408, Cdh1a=1.0884, Pai=0.49116, Pa=0.38889, Cb=0.078423, Mbi=0.080046, Mb=0.0097357, Mbp27=8.9043e-05, Cdc20i=0.7109, Cdc20a=0.010715, Pbi=0.55065, Pb=0.098727, Mw=0.017414, Wee1=0.41881, Wee1p=0.22374.

Fig.3f Mp=2.2664, Mc=3.0602, Mbmal=5.5945, Pc=0.014097, Cc=336.1068, Pcp=0.0081348, Ccp=0.7616, PCc=2.8863, PCn=1.421, PCcp=6.6519, PCnp=1.3352, Bc=1.7368, Bcp=0.10665, Bn=0.64466, Bnp=0.048635, In=0.78818, Mr=1.4611, Rc=0.47082, Rn=0.59183, AP1=6.0606, pRB=1.4098, pRBc1=2.5732, pRBp=12.392, pRBc2=11.3029, pRBpp=0.0015619, E2F=18.3528, E2Fp=0.016924, Cd=0.094145, Mdi=0.021792, Md=0.84884, Mdp27=0.49618, Mce=0.0052342, Ce=0.0028096, Mei=0.0083613, Me=0.03108, Skp2=21.094, Mep27=0.011245, Pei=0.15506, Pe=1.4204, Ca=0.0057082, Mai=0.020964, Ma=0.0067488, Map27=0.0014917, p27=0.17749, p27p=0.019447, Cdh1i=0.54031, Cdh1a=0.018351, Pai=0.58841, Pa=0.22268, Cb=0.043767, Mbi=0.035965, Mb=0.2511, Mbp27=0.028654, Cdc20i=0.060229, Cdc20a=1.8437, Pbi=0.12412, Pb=0.95896, Mw=0.012206, Wee1=0.15976, Wee1p=0.22679.

Figs. 4b, 5a and Fig. S3(a): Mp=1.1569999, Mc=1.7789, Mbmal=7.2195001, Pc=0.0062671001, Cc=325.28311, Pcp=0.0034558999, Ccp=0.76152998, PCc=0.70789999, PCn=0.19575, PCcp=5.2571998, PCnp=0.12734, Bc=2.1372001, Bcp=0.11593, Bn=1.3151, Bnp=0.076318003, In=0.055312, Mr=0.96881002, Rc=0.1548, Rn=0.041343, AP1=6.0605998, pRB=1.5329, pRBc1=0.48471999, pRBp=12.9828, pRBc2=2.0545001, pRBpp=0.13319001, E2F=3.2801001, E2Fp=0.026215, Cd=0.094091997, Mdi=0.022845, Md=1.325, Mdp27=0.013466, Mce=0.024901001, Ce=0.005537, Mei=0.031500999, Me=1.1559, Skp2=11.8677, Mep27=0.0081121, Pei=0.014376, Pe=1.7049, Ca=0.0046791998, Mai=0.046822, Ma=0.019317999, Map27=0.0002039, p27=0.0035139001, p27p=0.019300999, Cdh1i=0.54710001, Cdh1a=0.0055264998, Pai=0.63407999, Pa=0.29585001, Cb=0.95919001, Mbi=0.034823999, Mb=0.45019999, Mbp27=0.00094055, Cdc20i=0.026786, Cdc20a=1.8816, Pbi=0.057624999, Pb=1.0843, Mw=0.016368, Wee1=0.13579001, Wee1p=0.32049.

Fig. 4c and Figs. S3(b) and S3(d): Mp=1.0568, Mc=1.8480999, Mbmal=6.3362999, Pc=0.0063677998, Cc=331.87189, Pcp=0.0036138999, Ccp=0.76160997, PCc=1.1508, PCn=0.53987998, PCcp=6.4289999, PCnp=0.9738, Bc=1.7359, Bcp=0.10505, Bn=0.78301001, Bnp=0.054529, In=0.13778999, Mr=0.80400997, Rc=0.12732001, Rn=0.056669999, AP1=6.0605998, pRB=1.6623, pRBc1=0.064092003, pRBp=14.2593, pRBc2=0.27438, pRBpp=0.073599003, E2F=0.38652, E2Fp=0.13256, Cd=0.094083004, Mdi=0.022838, Md=1.3206, Mdp27=0.017968001 Mce=0.043490998, Ce=0.0096067004, Mei=0.029376, Me=0.85652, Skp2=10.3268, Mep27=0.0081107998, Pei=0.01898, Pe=1.6959, Ca=0.0052708001, Mai=0.058660999, Ma=0.57453001, Map27=0.0034302, p27=0.0047358, p27p=0.019261001, Cdh1i=0.54900002, Cdh1a=0.001937, Pai=0.049509, Pa=1.3054, Cb=1.5501, Mbi=0.034116, Mb=0.45583999, Mbp27=0.0012757001, Cdc20i=0.026214, Cdc20a=1.6991, Pbi=0.056650002, Pb=1.0693001, Mw=0.012853, Wee1=0.11596, Wee1p=0.26273999.

Fig. 4d, 5c and Fig. S3(c): Mp=3.8206, Mc=4.4591999, Mbmal=7.0496998, Pc=0.022822, Cc=332.95831, Pcp=0.013212, Ccp=0.76144999, PCc=4.0913, PCn=1.4298, PCcp=5.9698, PCnp=0.91716999, Bc=2.6282001, Bcp=0.12933999, Bn=1.1401, Bnp=0.072456002, In=1.7034, Mr=2.4809999, Rc=1.4744999, Rn=1.2787, AP1=6.0605998, pRB=1.5723, pRBc1=10.3337, pRBp=12.772, pRBc2=42.0023, pRBpp=0.72290999, E2F=65.723701, E2Fp=0.026629999, Cd=0.094283, Mdi=0.022849999 Md=1.3284, Mdp27=0.010303 Mce=0.012209, Ce=6.1146002, Mei=0.093232997, Me=1.8104, Skp2=0.11376, Mep27=0.0086570997, Pei=0.0093919998, Pe=1.7142, Ca=2.9912, Mai=0.069659002, Ma=0.0089255003, Map27=2.6543001e-05, p27=0.0023914999, p27p=0.047150999, Cdh1i=0.001224, Cdh1a=1.0976, Pai=0.58535999, Pa=0.22962999, Cb=0.1178, Mbi=0.11398, Mb=0.0083007999, Mbp27=1.1417e-05, Cdc20i=0.71063, Cdc20a=0.0089766998, Pbi=0.55146003, Pb=0.096436001, Mw=0.02376, Wee1=0.74412, Wee1p=0.29271001.

Fig. 7a,b, Fig. S2(b) and Fig. S4: Mp=2.4993, Mc=3.0172, Mbmal=9.1559, Pc=0.014313, Cc=315.34851, Pcp=0.0080521004, Ccp=0.76121002, PCc=1.7591, PCn=0.32752001, PCcp=3.6020999, PCnp=0.14058, Bc=3.3485, Bcp=0.1399, Bn=2.4319999, Bnp=0.1055, In=0.22218999, Mr=1.8796, Rc=0.50357997, Rn=0.16938999, AP1=6.0605998, pRB=2.0323999, pRBc1=2.8015001, pRBp=15.9921, pRBc2=11.0375, pRBpp=0.17736, E2F=13.3293, E2Fp=0.57766998, Cd=0.094108999, Mdi=0.022846, Md=1.3261, Mdp27=0.012429, Mce=0.0025329001, Ce=0.0020113001, Mei=0.039349001, Me=1.2926, Skp2=3.2623, Mep27=0.0081824996, Pei=0.012941, Pe=1.7078, Ca=0.10244, Mai=0.077680998, Ma=0.24383, Map27=0.00087791, p27=0.0031552999, p27p=0.019941, Cdh1i=0.54447001, Cdh1a=0.013721, Pai=0.12989999, Pa=1.1174001, Cb=0.83148003, Mbi=0.29302001, Mb=0.044936001, Mbp27=7.1399998e-05, Cdc20i=0.67309999, Cdc20a=0.076699004, Pbi=0.50858998, Pb=0.1675, Mw=0.01198, Wee1=0.24507999, Wee1p=0.1787

Fig. 7e,f and Fig. S2(c): Mp=2.1944001, Mc=2.7155001, Mbmal=8.7518997, Pc=0.01238, Cc=316.4129, Pcp=0.0069275, Ccp=0.76126999, PCc=1.4507, PCn=0.27621001, PCcp=3.7061999, PCnp=0.11497, Bc=3.0973001, Bcp=0.13585, Bn=2.2598, Bnp=0.102, In=0.15332, Mr=1.6896, Rc=0.39939001, Rn=0.12418, AP1=6.0605998, pRB=1.8511, pRBc1=3.49, pRBp=14.8012, pRBc2=13.9825, pRBpp=0.16383, E2F=18.4433, E2Fp=0.5887, Cd=0.094125003, Mdi=0.022843, Md=1.3241, Mdp27=0.014482, Mce=0.0040384, Ce=0.0048019998, Mei=0.040137, Me=1.2557, Skp2=1.4085, Mep27=0.0082513001, Pei=0.013299, Pe=1.7065001, Ca=0.15322, Mai=0.088518001, Ma=0.23305, Map27=0.00077024999, p27=0.0032674, p27p=0.021024, Cdh1i=0.57270998, Cdh1a=0.018758001, Pai=0.13643, Pa=1.0465, Cb=0.38834, Mbi=0.17692, Mb=0.023291999, Mbp27=3.5796002e-05, Cdc20i=0.69796002, Cdc20a=0.029544, Pbi=0.53530002, Pb=0.12092, Mw=0.01198, Wee1=0.25659001, Wee1p=0.17239

Fig. 7c,d and Fig. S2(d): Mp=2.3504, Mc=3.1379001, Mbmal=5.5969, Pc=0.014588, Cc=336.16791, Pcp=0.0084201004, Ccp=0.76159, PCc=2.9741001, PCn=1.4513, PCcp=6.6378999, PCnp=1.3285, Bc=1.7568001, Bcp=0.10734, Bn=0.65192997, Bnp=0.049095999, In=0.83809, Mr=1.5125, Rc=0.51437998, Rn=0.64007998, AP1=6.0605998, pRB=1.5642, pRBc1=11.2912, pRBp=12.1498, pRBc2=43.853199, pRBpp=1.408, E2F=72.184402, E2Fp=0.0053323, Cd=0.094313003, Mdi=0.022852, Md=1.3297, Mdp27=0.0089892, Mce=0.046331, Ce=18.3297, Mei=0.041972999, Me=1.9401, Skp2=0.11374, Mep27=0.0087379003, Pei=0.0087884003, Pe=1.7158, Ca=2.4066999, Mai=0.0062527, Ma=0.0017703, Map27=5.0844001e-06, p27=0.0022541001, p27p=0.047812998, Cdh1i=0.00091857999, Cdh1a=1.0982, Pai=0.59601998, Pa=0.20821001, Cb=0.068705998, Mbi=0.071603, Mb=0.011518, Mbp27=1.5565e-05, Cdc20i=0.70967001, Cdc20a=0.012924, Pbi=0.54922003, Pb=0.10155, Mw=0.01198, Wee1=0.26192999, Wee1p=0.16855

**7. Figures S1 to S7**

**Figure S1:** Birhythmicity in conditions where the cell cycle is coupled to the circadian clock only via cyclin E. (a) The cell cycle can be entrained to 24h (left region) or 48h (right region) depending on the coupling strength measured by parameter *v*_sce_ and on the autonomous period of the cell cycle prior to its coupling to the circadian clock [32]. Birhythmicity is observed in each of the two domains of entrainment, in the indicated area above the red and blue lines, respectively. (b,c) Time evolution of cyclin B/Cdk1 and cyclin E/Cdk2 when the autonomous period of the cell cycle is 19.53h, and the coupling strength *v*_sce_ is 0.1 μMh^-1^. The cell cycle is entrained to 24h (b) or exhibits very low amplitude oscillations (note the reduced scale) of 24h-period (c), depending on initial conditions (see Supporting Informations, Section 6). The autonomous period of the cell cycle is obtained by setting in (b) and (c) the value of the scaling parameter *eps* = 22. (d-e) Time evolution of cyclin B/Cdk1 and cyclin E/Cdk2 when the autonomous period of the cell cycle is 39.8h, and the coupling strength *v*_ce_ is 0.1. The cell cycle is entrained to 48h (d) or exhibits very low amplitude oscillations with a period of 24h (e), depending on initial conditions. The autonomous period is obtained by setting in (d) and (e) the value of the scaling parameter *eps* = 10.8.

**Figure S2:** Trirhythmicity in conditions where the cell cycle is coupled to the circadian clock only via cyclin E. (a) The cell cycle can be entrained to 24 or 48 h, as shown in figure 3. Trirhythmicity is observed in the narrow area in green in the domain of birhythmicity on the left. (b–d) Time evolution of cyclin B/Cdk1 and cyclin E/Cdk2 when the autonomous period of the cell cycle is 19.36 h, and the coupling strength (*v*_sce_) is equal to 0.06 μMh^−1^. The cell cycle is entrained to 24 h (b), exhibits multiple large peaks in a cycle of 72 h-period (c) or low-amplitude oscillations (note the reduced scale) of 24 h-period (d), depending on initial conditions. The value of the scaling parameter *eps* is 22.2 in (b–d).

**Figure S3:** Trirhythmicity: switching between multiple periodic attractors when the cell cycle is coupled to the circadian clock via both Wee1 and cyclin E, in the conditions of Fig. 4b-d. (a) Switch from simple oscillations of 24h-period to complex oscillations of 48h-period when the basal synthesis rate of cyclin B (*v*_cb_) transiently decreases from 0.055μMh^-1^ to 0.005 μMh^-1^ at t=480h (arrow); as in all panels in this figure, the transient change in *v*_cb_ lasts for 1h. (b) Complex oscillations can also switch back to simple oscillations when *v*_cb_ decreases transiently from 0.055 μMh^-1^ to 0.005 μMh^-1^ at t=480h. (c) Endoreplication can switch to complex oscillations when *v*_cb_ increases from 0.055 μMh^-1^ to 0.5 μMh^-1^ at t=478h. (d) Complex oscillations can also switch back to endoreplication when *v*_cb_ increases from 0.055 μMh^-1^ to 1 μMh^-1^ at t=455h. The autonomous period of the cell cycle is 20.08h (*eps*=21.4) and the coupling strength *μ*=0.05.

**Figure S4:** Sensitivity to duration of the transient perturbation for switching between multiple periodic regimes. In the conditions of Fig 7b, when *v*_cb_ increases transiently from 0.055 μMh^-1^ to 0.5 μMh^-1^ at t=480h (arrow), simple oscillations fail to switch to another periodic attractor if the duration of the change is 0.1h-0.3h, 0.7-0.8h, 1.4-1.5h, or 1.7-1.9h. If the duration of the change is 1h-1.2h, simple oscillations will switch to endoreplication [(a), duration=1h]. If the duration of the change is 0.4h-0.6h, 0.9h, 1.3h, 1.6h and 2h, simple oscillations will switch to complex oscillation [(b), duration=0.4h]. Initial conditions are as in Fig. 7b.

**Figure S5:** Sensitivity to magnitude of the transient perturbation for switching between multiple periodic regimes. In the condition of Fig. S3(a), at t=480h (arrow), if the magnitude of *v*_cb_ transiently changes for 1h, simple oscillations may switch to another periodic attractor. If the value of *v*_cb_ is increased up to 0.026-0.064, 0.954-1.57, 1.584-1.595 or 1.598-1.599, simple oscillations fail to switch. If *v*_cb_ is transiently changed to 0-0.025, 0.065-0.953, 1.571-1.583 or 1.596-1.597, simple oscillations switch to complex oscillations [(a), magnitude =0.005]. If the magnitude is increased to 1.6-2, simple oscillations switch to endoreplication [(b), magnitude =1.6]. Initial conditions are as in Fig. S3(a).

**Figure S6:** Parameters other than *v*_cb_ can also cause the transition between multiple periodic attractors. (a) In the same initial conditions as in Fig. 7e if the total Cdk2 (Cdk2_tot_) is decreased from 2 μM to 1 μM at t=480h for 1 hour, the complex oscillation can switch to simple oscillation of 24h. (b) If the basal rate of Wee1 mRNA (*v_swee1_*) synthesis increases at t=480h from 0.0117μMh^-1^ to 0.05 μMh^-1^ for 1 hour, the complex oscillations can also change to simple oscillations. (c) With the same initial conditions as in Fig. 7a the simple oscillations can switch back to complex oscillations by decreasing the total Cdk1 (Cdk1_tot_) from 0.5 μM to 0.1 μM for 1 hour at t=485h.

**Figure S7:** Bifurcation scenario for the onset of birhythmicity and trirhythmicity. The schematic bifurcation diagram shows the origin of multiple periodic attractors in a 3-variable model for a multiply regulated biochemical system [28]. The solid lines represents the stable oscillatory regimes (limit cycles LC1, LC2 or LC3) or the stable steady state of one of the variables, denoted X_0_. The dashed line indicates an unstable steady state or unstable periodic trajectories. As the control parameter increases, the coupled system could switch from a single periodic solution (LC1) to birhythmicity (coexistence of LC1 and LC2) and trirhythmicity (coexistence of LC1, LC2 and LC3). The relevance of this bifurcation diagram to the occurrence of multirhythmicity in the coupled circadian clock-cell cycle model remains to be established; this question might be investigated more easily by means of reduced models for the two cellular oscillators. The results shown in Fig. S7 suggest that the coupling strength can play the role of control parameter whose increase leads successively from monorhythmicity to birhythmicity and trirhythmicity.

**8. References**

1. Gérard C, Goldbeter A. 2009 Temporal self-organization of the cyclin/Cdk network driving the mammalian cell cycle. *Proc. Natl Acad. Sci. USA* **106**, 21643-21648.

2. Leloup JC, Goldbeter A. 2003 Toward a detailed computational model for the mammalian circadian clock. *Proc. Natl Acad. Sci. USA* **100,** 7051-7056.

3. Leloup JC, Goldbeter A. 2004 Modeling the mammalian circadian clock : Sensitivity analysis and multiplicity of oscillatory mechanisms. *J. Theor. Biol.* **230**, 541-562.

4. Gérard C, Goldbeter A. 2012 Entrainment of the mammalian cell cycle by the circadian clock : Modeling two coupled cellular rhythms. *PLoS Comput. Biol.* **8**, e1002516.

5. Morgan DO. 2006 *The cell cycle: Principles of control*. Oxford, UK: Oxford University Press.

6. Matsuo T, Yamaguchi S, Mitsui S, Emi A, Shimoda F, Okamura H. 2003 Control mechanism of the circadian clock for timing of cell division in vivo. *Science* **302**, 255-259.

7. Fu L, Pelicano H, Liu J, Huang P, Lee CC. 2002 The circadian gene *Period2* plays an important role in tumor suppression and DNA damage response in vivo. *Cell* **111**, 41-50.

8. Pérez-Roger I, Solomon DLC, Sewing A, Land H. 1997 Myc activation of cyclin E/Cdk2 kinase involves induction of cyclin E gene transcription and inhibition of p27^Kip1^ binding to newly formed complexes. *Oncogene* **14**, 2373-2381.

9. Ermentrout B. 2007 XPPAUT. *Scholarpedia* **2**(1), 1399.

**9. Computer code used in numerical simulations of the model for the coupling of
the cell cycle to the circadian clock**

**9.1 Coupling the cell cycle to the circadian clock via Wee1 only**

Mp'=v_sP*Bn^n/(K_AP^n+Bn^n)-v_mP*Mp/(K_mP+Mp)-k_dmp*Mp

Mc'=v_sC*Bn^n/(K_AC^n+Bn^n)-v_mC*Mc/(K_mC+Mc)-k_dmc*Mc

Mbmal'=v_sB*K_IB^m/(K_IB^m+Rn^m)-v_mB*Mbmal/(K_mB+Mbmal)-k_dmb*Mbmal

Pc'=k_sP*Mp-V_1P*Pc/(K_p+Pc)+V_2P*Pcp/(K_dp+Pcp)+k4_clock*PCc-k3_clock*Pc*Cc-k_dn*Pc

Cc'=k_sC*Mc-V_1C*Cc/(K_p+Cc)+V_2C*Ccp/(K_dp+Ccp)+k4_clock*PCc-k3_clock*Pc*Cc-k_dnc*Cc

Pcp'=V_1P*Pc/(K_p+Pc)-V_2P*Pcp/(K_dp+Pcp)-v_dPC*Pcp/(K_d+Pcp)-k_dn*Pcp

Ccp'=V_1C*Cc/(K_p+Cc)-V_2C*Ccp/(K_dp+Ccp)-v_dCC*Ccp/(K_d+Ccp)-k_dn*Ccp

PCc'=-V_1PC*PCc/(K_p+PCc)+V_2PC*PCcp/(K_dp+PCcp)-k4_clock*PCc+k3_clock*Pc*Cc+\

k2_clock*PCn-k1_clock*PCc-k_dn*PCc

PCn'=-V_3PC*PCn/(K_p+PCn)+V_4PC*PCnp/(K_dp+PCnp)-k2_clock*PCn+k1_clock*PCc-k7*Bn\

*PCn+k8*In-k_dn*PCn

PCcp'=V_1PC*PCc/(K_p+PCc)-V_2PC*PCcp/(K_dp+PCcp)-v_dPCC*PCcp/(K_d+PCcp)-k_dn*PCcp

PCnp'=V_3PC*PCn/(K_p+PCn)-V_4PC*PCnp/(K_dp+PCnp)-v_dPCN*PCnp/(K_d+PCnp)-k_dn*PCnp

Bc'=k_sB*Mbmal-V_1B*Bc/(K_p+Bc)+V_2B*Bcp/(K_dp+Bcp)-k5*Bc+k6*Bn-k_dn*Bc

Bcp'=V_1B*Bc/(K_p+Bc)-V_2B*Bcp/(K_dp+Bcp)-v_dBC*Bcp/(K_d+Bcp)-k_dn*Bcp

Bn'=-V_3B*Bn/(K_p+Bn)+V_4B*Bnp/(K_dp+Bnp)+k5*Bc-k6*Bn-k7*Bn*PCn+k8*In-k_dn*Bn

Bnp'=V_3B*Bn/(K_p+Bn)-V_4B*Bnp/(K_dp+Bnp)-v_dBN*Bnp/(K_d+Bnp)-k_dn*Bnp

In'=-k8*In+k7*Bn*PCn-v_dIN*In/(K_d+In)-k_dn*In

Mr'=v_sR*Bn^h/(K_AR^h+Bn^h)-v_mR*Mr/(K_mR+Mr)-k_dmr*Mr

Rc'=k_sR*Mr-k9*Rc+k10*Rn-v_dRC*Rc/(K_d+Rc)-k_dn*Rc

Rn'=k9*Rc-k10*Rn-v_dRN*Rn/(K_d+Rn)-k_dn*Rn

#cell cycle

AP1'=(v_sap1*(GF/(K_agf+GF))-k_dap1*AP1)*eps

pRB'=(v_sprb-k_pc1*pRB*E2F+k_pc2*pRBc1-V1*(pRB/(K1+pRB))*(Md+Mdp27)+V2*(pRBp/(K2+pRBp))\

-k_dprb*pRB)*eps

pRBc1'=(k_pc1*pRB*E2F-k_pc2*pRBc1)*eps

pRBp'=(V1*(pRB/(K1+pRB))*(Md+Mdp27)-V2*(pRBp/(K2+pRBp))-V3*(pRBp/(K3+pRBp))*Me+\

V4*(pRBpp/(K4+pRBpp))-k_pc3*pRBp*E2F+k_pc4*pRBc2-k_dprbp*pRBp)*eps

pRBc2'=(k_pc3*pRBp*E2F-k_pc4*pRBc2)*eps

pRBpp'=(V3*(pRBp/(K3+pRBp))*Me-V4*(pRBpp/(K4+pRBpp))-k_dprbpp*pRBpp)*eps

E2F'=(v_se2f-k_pc1*pRB*E2F+k_pc2*pRBc1-k_pc3*pRBp*E2F+k_pc4*pRBc2-V_1e2f*Ma*\

(E2F/(K_1e2f+E2F))+V_2e2f*(E2Fp/(K_2e2f+E2Fp))-k_de2f*E2F)*eps

E2Fp'=(V_1e2f*Ma*(E2F/(K_1e2f+E2F))-V_2e2f*(E2Fp/(K_2e2f+E2Fp))-k_de2fp*E2Fp)*eps

# Module Cyclin D/Cdk4-6 : G1 phase

Cd'=(k_cd1*AP1+k_cd2*E2F*(K_i7/(K_i7+pRB))*(K_i8/(K_i8+pRBp))-k_com1*Cd*\

(Cdk4_tot-(Mdi+Md+Mdp27))+k_decom1*Mdi-V_dd*(Cd/(K_dd+Cd))-k_ddd*Cd)*eps

Mdi'=(k_com1*Cd*(Cdk4_tot-(Mdi+Md+Mdp27))-k_decom1*Mdi+V_m2d*(Md/(K_2d+Md))-\

V_m1d*(Mdi/(K_1d+Mdi)))*eps

Md'=(V_m1d*(Mdi/(K_1d+Mdi))-V_m2d*(Md/(K_2d+Md))-k_c1*Md*p27+k_c2*Mdp27)*eps

Mdp27'=(k_c1*Md*p27-k_c2*Mdp27)*eps

#Module Cyclin E/Cdk2: G1 phase and transition G1/S

Mce'=v_sce*K_ice^nce/(K_ice^nce+Bn^nce)-V_dmce*Mce/(K_dmce+Mce)

Ce'=(k_ce*E2F*(K_i9/(K_i9+pRB))*(K_i10/(K_i10+pRBp))+k_ce2*Mce-k_com2*Ce*\

(Cdk2_tot-(Mei+Me+Mep27+Mai+Ma+Map27))+k_decom2*Mei-V_de*(Skp2/(K_dceskp2+Skp2))*\

(Ce/(K_de+Ce))-k_dde*Ce)*eps

Mei'=(k_com2*Ce*(Cdk2_tot-(Mei+Me+Mep27+Mai+Ma+Map27))-k_decom2*Mei+V_m2e*(Wee1+i_b1)\

*(Me/(K_2e+Me))-V_m1e*Pe*(Mei/(K_1e+Mei)))*eps

Me'=(V_m1e*Pe*(Mei/(K_1e+Mei))-V_m2e*(Wee1+i_b1)*(Me/(K_2e+Me))-k_c3*Me*p27+k_c4*Mep27)*eps

Skp2'=(v_sskp2-V_dskp2*(Skp2/(K_dskp2+Skp2))*(Cdh1a/(K_cdh1+Cdh1a))-k_ddskp2*Skp2)*eps

Mep27'=(k_c3*Me*p27-k_c4*Mep27)*eps

Pei'=(v_spei+V_6e*(x_e1+x_e2*Chk1)*(Pe/(K_6e+Pe))-V_m5e*(Me+a_e)*(Pei/(K_5e+Pei))\

-k_dpei*Pei)*eps

Pe'=(V_m5e*(Me+a_e)*(Pei/(K_5e+Pei))-V_6e*(x_e1+x_e2*Chk1)*(Pe/(K_6e+Pe))-k_dpe*Pe)*eps

# Module Cyclin A/Cdk2 : S phase and transition S/G2

Ca'=(k_ca*E2F*(K_i11/(K_i11+pRB))*(K_i12/(K_i12+pRBp))-k_com3*Ca*\

(Cdk2_tot-(Mei+Me+Mep27+Mai+Ma+Map27))+\

k_decom3*Mai-V_da*(Ca/(K_da+Ca))*(Cdc20a/(K_acdc20+Cdc20a))-k_dda*Ca)*eps

Mai'=(k_com3*Ca*(Cdk2_tot-(Mei+Me+Mep27+Mai+Ma+Map27))-k_decom3*Mai+V_m2a*(Wee1+i_b2)\

*(Ma/(K_2a+Ma))-V_m1a*Pa*(Mai/(K_1a+Mai)))*eps

Ma'=(V_m1a*Pa*(Mai/(K_1a+Mai))-V_m2a*(Wee1+i_b2)*(Ma/(K_2a+Ma))-k_c5*Ma*p27+k_c6*Map27)*eps

Map27'=(k_c5*Ma*p27-k_c6*Map27)*eps

p27'=(v_s1p27+v_s2p27*E2F*(K_i13/(K_i13+pRB))*(K_i14/(K_i14+pRBp))-k_c1*Md*p27+k_c2*Mdp27\

-k_c3*Me*p27+k_c4*Mep27-k_c5*Ma*p27+k_c6*Map27\

-k_c7*Mb*p27+k_c8*Mbp27-V_1p27*Me*(p27/(K_1p27+p27))+V_2p27*(p27p/(K_2p27+p27p))\

-k_ddp27*p27)*eps

p27p'=(V_1p27*Me*(p27/(K_1p27+p27))-V_2p27*(p27p/(K_2p27+p27p))-V_dp27p*\

(Skp2/(K_dp27skp2+Skp2))*(p27p/(K_dp27p+p27p))-k_ddp27p*p27p)*eps

Cdh1i'=(V_2cdh1*(Cdh1a/(K_2cdh1+Cdh1a))*(Ma+Mb)-V_1cdh1*(Cdh1i/(K_1cdh1+Cdh1i))-\

k_dcdh1i*Cdh1i)*eps

Cdh1a'=(v_scdh1a+V_1cdh1*(Cdh1i/(K_1cdh1+Cdh1i))-V_2cdh1*(Cdh1a/(K_2cdh1+Cdh1a))*\

(Ma+Mb)-k_dcdh1a*Cdh1a)*eps

Pai'=(v_spai+V_6a*(x_a1+x_a2*Chk1)*(Pa/(K_6a+Pa))-V_m5a*(Ma+a_a)*(Pai/(K_5a+Pai))-\

k_dpai*Pai)*eps

Pa'=(V_m5a*(Ma+a_a)*(Pai/(K_5a+Pai))-V_6a*(x_a1+x_a2*Chk1)*(Pa/(K_6a+Pa))-k_dpa*Pa)*eps

# Module Cyclin B/Cdk1 : G2 phase and transition G2/M

Cb'=(v_cb-k_com4*Cb*(Cdk1_tot-(Mbi+Mb+Mbp27))+k_decom4*Mbi-V_db*(Cb/(K_db+Cb))*\

((Cdc20a/(K_dbcdc20+Cdc20a))+(Cdh1a/(K_dbcdh1+Cdh1a)))-k_ddb*Cb)*eps

Mbi'=(k_com4*Cb*(Cdk1_tot-(Mbi+Mb+Mbp27))-k_decom4*Mbi+V_m2b*(Wee1+i_b3)*(Mb/(K_2b+Mb))\

-V_m1b*Pb*(Mbi/(K_1b+Mbi)))*eps

Mb'=(V_m1b*Pb*(Mbi/(K_1b+Mbi))-V_m2b*(Wee1+i_b3)*(Mb/(K_2b+Mb))-k_c7*Mb*p27+k_c8*Mbp27)*eps

Mbp27'=(k_c7*Mb*p27-k_c8*Mbp27)*eps

Cdc20i'=(v_scdc20i-V_m3b*Mb*(Cdc20i/(K_3b+Cdc20i))+V_m4b*(Cdc20a/(K_4b+Cdc20a))\

-k_dcdc20i*Cdc20i)*eps

Cdc20a'=(V_m3b*Mb*(Cdc20i/(K_3b+Cdc20i))-V_m4b*(Cdc20a/(K_4b+Cdc20a))-k_dcdc20a*Cdc20a)*eps

Pbi'=(v_spbi+V_6b*(x_b1+x_b2*Chk1)*(Pb/(K_6b+Pb))-V_m5b*(Mb+a_b)*\

(Pbi/(K_5b+Pbi))-k_dpbi*Pbi)*eps

Pb'=(V_m5b*(Mb+a_b)*(Pbi/(K_5b+Pbi))-V_6b*(x_b1+x_b2*Chk1)*(Pb/(K_6b+Pb))-k_dpb*Pb)*eps

#coupling

Mw'=v_swee1+v_sw*Bn^nmw/(K_aw^nmw+Bn^nmw)-V_dmw*Mw/(K_dmw+Mw)

Wee1'=(k_sw*Mw-V_m7b*(Mb+i_b)*(Wee1/(K_7b+Wee1))+V_m8b*(Wee1p/(K_8b+Wee1p))-k_dwee1*Wee1)*eps

Wee1p'=(V_m7b*(Mb+i_b)*(Wee1/(K_7b+Wee1))-V_m8b*(Wee1p/(K_8b+Wee1p))-k_dwee1p*Wee1p)*eps

#parameters

par k1_clock=0.8,k2_clock=0.4,k3_clock=0.8,k4_clock=0.4,k5=0.8,k6=0.4,k7=1,k8=0.2,k9=0.8,k10=0.4,\

K_AP=0.6,K_AC=0.6,K_AR=0.6,K_IB=1,\

k_dmb=0.02,k_dmc=0.02,k_dmp=0.02,k_dmr=0.02,k_dn=0.02,k_dnc=0.02,\

K_d=0.3,K_dp=0.1,K_p=1.006,K_mB=0.4,K_mC=0.4,K_mP=0.3,K_mR=0.4,k_sB=0.32,\

k_sC=3.2,k_sP=1.2,k_sR=1.7,m=2,h=2,n=2,\

V_1B=1.4,V_1C=1.2,V_1P=9.6,V_1PC=2.4,V_2B=0.2,V_2C=0.2,V_2P=0.6,V_2PC=0.2,\

V_3B=1.4,V_3PC=2.4,V_4B=0.4,V_4PC=0.2,V_phos=0.4,v_dBC=3,v_dBN=3,v_dCC=1.4,\

v_dIN=1.6,v_dPC=3.4,v_dPCC=1.4,v_dPCN=1.4,v_dRC=4.4,v_dRN=0.8,v_mB=1.3,\

v_mC=2.0,v_mP=2.2,v_mR=1.6,v_sB=1.8,\

v_sC=2.2,v_sP=2.4,v_sR=1.6

#Cell cycle

par Chk1=0,u=1

par GF=1,K_agf=0.1,k_dap1=0.15,eps=13,v_sap1=1

par k_de2f=0.002,k_de2fp=1.1,k_dprb=0.01,k_dprbp=0.06,k_dprbpp=0.04

par k_pc1=0.05,k_pc2=0.5,k_pc3=0.025,k_pc4=0.5,K1=0.1,K2=0.1,K3=0.1

par K4=0.1,V1=2.2,V2=2,V3=1,V4=2,K_1e2f=5,K_2e2f=5,V_1e2f=4

par V_2e2f=0.75,v_se2f=0.15,v_sprb=0.8

par Cdk4_tot=1.5,K_i7=0.1,K_i8=2,k_cd1=0.4,k_cd2=0.005,k_decom1=0.1

par k_com1=0.175,k_c1=0.15,k_c2=0.05,k_ddd=0.005,K_dd=0.1,K_1d=0.1,K_2d=0.1

par V_dd=5,V_m1d=1,V_m2d=0.2

par a_e=0.25,Cdk2_tot=2,i_b1=0.5,K_i9=0.1,K_i10=2,k_ce=0.29,k_c3=0.2

par k_c4=0.1,k_decom2=0.1,k_com2=0.2,k_dde=0.005,k_ddskp2=0.005,k_dpe=0.075

par k_dpei=0.15,K_de=0.1,K_dceskp2=2,K_dskp2=0.5,K_cdh1=0.4,K_1e=0.1

par K_2e=0.1,K_5e=0.1,K_6e=0.1,V_de=3,V_dskp2=1.1,V_m1e=2,V_m2e=1.4,V_m5e=5

par V_6e=0.8,v_spei=0.13,v_sskp2=0.15,x_e1=1,x_e2=1

par a_a=0.2,i_b2=0.5,K_i11=0.1,K_i12=2,K_i13=0.1,K_i14=2,k_ca=0.0375

par k_decom3=0.1,k_com3=0.2,k_c5=0.15,k_c6=0.125,k_dda=0.005,k_ddp27=0.06

par k_ddp27p=0.01,k_dcdh1a=0.1,k_dcdh1i=0.2,k_dpa=0.075,k_dpai=0.15,K_da=1.1

par K_dp27p=0.1,K_dp27skp2=0.1,K_acdc20=2,K_1a=0.1,K_2a=0.1,K_1cdh1=0.01

par K_2cdh1=0.01,K_5a=0.1,K_6a=0.1,K_1p27=0.5,K_2p27=0.5,V_dp27p=5,V_da=2.5

par V_m1a=2,V_m2a=1.85,V_m5a=4,V_6a=1,v_scdh1a=0.11,v_spai=0.105

par v_s1p27=0.8,v_s2p27=0.1,V_1cdh1=1.25,V_2cdh1=8,V_1p27=100,V_2p27=0.1

par x_a1=1,x_a2=1

par a_b=0.11,Cdk1_tot=0.5,i_b=0.75,i_b3=0.5,k_c7=0.12,k_c8=0.2

par k_decom4=0.1,k_com4=0.25,k_dcdc20a=0.05,k_dcdc20i=0.14,k_ddb=0.005

par k_dpb=0.1,k_dpbi=0.2,k_dwee1=0.1,k_dwee1p=0.2,K_db=0.005,K_dbcdc20=0.2,K_dbcdh1=0.1

par k_sw=5,K_1b=0.1,K_2b=0.1,K_3b=0.1,K_4b=0.1,K_5b=0.1,K_6b=0.1,K_7b=0.1

par K_8b=0.1,v_cb=0.055,V_db=0.06,V_m1b=3.9,V_m2b=2.1,v_scdc20i=0.1,V_m3b=8,V_m4b=0.7

par V_m5b=5,V_6b=1,V_m7b=1.2,V_m8b=1,v_spbi=0.12,x_b1=1,x_b2=1

#coupling

par v_sw=0.0146,v_swee1=0.0117,nmw=4,K_aw=2,V_dmw=0.5,K_dmw=0.5

par v_sce=0,K_ice=1,V_dmce=0.5,K_dmce=0.5,nce=4,k_ce2=5

#some initial conditions

init Mp=4.0342002,Mc=4.6187,Mbmal=8.4909,Pc=0.023964999,Cc=327.7673,\

Pcp=0.013851,Ccp=0.76131999,PCc=3.9844,PCn=1.0813,PCcp=5.3548002

init PCnp=0.62621999,Bc=3.3185,Bcp=0.14084999,Bn=1.7053,Bnp=0.090561002,\

In=1.6584001,Mr=2.6716001,Rc=1.5322,Rn=1.1181999

#cell cycle

init AP1=6.0605998,pRB=1.4225,pRBc1=1.567,pRBp=12.2283,pRBc2=6.7241998,pRBpp=0.00099184003,E2F=11.1372,E2Fp=0.0077661001

init Cd=0.094114996,Mdi=0.021430001,Md=0.74659997,Mdp27=0.59710002

init Mce=0,Ce=0.0011064,Mei=0.0061730999,Me=0.019832,Skp2=21.161699,Mep27=0.012164,Pei=0.17065001,Pe=1.3978

init Ca=0.003338,Mai=0.011651,Ma=0.0035418,Map27=0.0011525,p27=0.32516,p27p=0.018523,Cdh1i=0.54514003,Cdh1a=0.0093810996,Pai=0.59320003,Pa=0.21314

init Cb=0.22935,Mbi=0.039067999,Mb=0.34164,Mbp27=0.062483002,Cdc20i=0.038474001,Cdc20a=1.8981,Pbi=0.081721999,Pb=1.0404,Mw=0.019308999,Wee1=0.20207

init Wee1p=0.39875999.

@ bounds=10000,total=1500,Method=Qualst.RK4,dt=0.01

@ runnow=1

done

**9.2 Coupling the cell cycle to circadian clock via cyclin E only**

Mp'=v_sP*Bn^n/(K_AP^n+Bn^n)-v_mP*Mp/(K_mP+Mp)-k_dmp*Mp

Mc'=v_sC*Bn^n/(K_AC^n+Bn^n)-v_mC*Mc/(K_mC+Mc)-k_dmc*Mc

Mbmal'=v_sB*K_IB^m/(K_IB^m+Rn^m)-v_mB*Mbmal/(K_mB+Mbmal)-k_dmb*Mbmal

Pc'=k_sP*Mp-V_1P*Pc/(K_p+Pc)+V_2P*Pcp/(K_dp+Pcp)+k4_clock*PCc-k3_clock*Pc*Cc-k_dn*Pc

Cc'=k_sC*Mc-V_1C*Cc/(K_p+Cc)+V_2C*Ccp/(K_dp+Ccp)+k4_clock*PCc-k3_clock*Pc*Cc-k_dnc*Cc

Pcp'=V_1P*Pc/(K_p+Pc)-V_2P*Pcp/(K_dp+Pcp)-v_dPC*Pcp/(K_d+Pcp)-k_dn*Pcp

Ccp'=V_1C*Cc/(K_p+Cc)-V_2C*Ccp/(K_dp+Ccp)-v_dCC*Ccp/(K_d+Ccp)-k_dn*Ccp

PCc'=-V_1PC*PCc/(K_p+PCc)+V_2PC*PCcp/(K_dp+PCcp)-k4_clock*PCc+k3_clock*Pc*Cc+\

k2_clock*PCn-k1_clock*PCc-k_dn*PCc

PCn'=-V_3PC*PCn/(K_p+PCn)+V_4PC*PCnp/(K_dp+PCnp)-k2_clock*PCn+k1_clock*PCc-k7*Bn\

*PCn+k8*In-k_dn*PCn

PCcp'=V_1PC*PCc/(K_p+PCc)-V_2PC*PCcp/(K_dp+PCcp)-v_dPCC*PCcp/(K_d+PCcp)-k_dn*PCcp

PCnp'=V_3PC*PCn/(K_p+PCn)-V_4PC*PCnp/(K_dp+PCnp)-v_dPCN*PCnp/(K_d+PCnp)-k_dn*PCnp

Bc'=k_sB*Mbmal-V_1B*Bc/(K_p+Bc)+V_2B*Bcp/(K_dp+Bcp)-k5*Bc+k6*Bn-k_dn*Bc

Bcp'=V_1B*Bc/(K_p+Bc)-V_2B*Bcp/(K_dp+Bcp)-v_dBC*Bcp/(K_d+Bcp)-k_dn*Bcp

Bn'=-V_3B*Bn/(K_p+Bn)+V_4B*Bnp/(K_dp+Bnp)+k5*Bc-k6*Bn-k7*Bn*PCn+k8*In-k_dn*Bn

Bnp'=V_3B*Bn/(K_p+Bn)-V_4B*Bnp/(K_dp+Bnp)-v_dBN*Bnp/(K_d+Bnp)-k_dn*Bnp

In'=-k8*In+k7*Bn*PCn-v_dIN*In/(K_d+In)-k_dn*In

Mr'=v_sR*Bn^h/(K_AR^h+Bn^h)-v_mR*Mr/(K_mR+Mr)-k_dmr*Mr

Rc'=k_sR*Mr-k9*Rc+k10*Rn-v_dRC*Rc/(K_d+Rc)-k_dn*Rc

Rn'=k9*Rc-k10*Rn-v_dRN*Rn/(K_d+Rn)-k_dn*Rn

#cell cycle

AP1'=(v_sap1*(GF/(K_agf+GF))-k_dap1*AP1)*eps

pRB'=(v_sprb-k_pc1*pRB*E2F+k_pc2*pRBc1-V1*(pRB/(K1+pRB))*(Md+Mdp27)+V2*(pRBp/(K2+pRBp))\

-k_dprb*pRB)*eps

pRBc1'=(k_pc1*pRB*E2F-k_pc2*pRBc1)*eps

pRBp'=(V1*(pRB/(K1+pRB))*(Md+Mdp27)-V2*(pRBp/(K2+pRBp))-V3*(pRBp/(K3+pRBp))*Me+\

V4*(pRBpp/(K4+pRBpp))-k_pc3*pRBp*E2F+k_pc4*pRBc2-k_dprbp*pRBp)*eps

pRBc2'=(k_pc3*pRBp*E2F-k_pc4*pRBc2)*eps

pRBpp'=(V3*(pRBp/(K3+pRBp))*Me-V4*(pRBpp/(K4+pRBpp))-k_dprbpp*pRBpp)*eps

E2F'=(v_se2f-k_pc1*pRB*E2F+k_pc2*pRBc1-k_pc3*pRBp*E2F+k_pc4*pRBc2-V_1e2f*Ma*\

(E2F/(K_1e2f+E2F))+V_2e2f*(E2Fp/(K_2e2f+E2Fp))-k_de2f*E2F)*eps

E2Fp'=(V_1e2f*Ma*(E2F/(K_1e2f+E2F))-V_2e2f*(E2Fp/(K_2e2f+E2Fp))-k_de2fp*E2Fp)*eps

# Module Cyclin D/Cdk4-6 : G1 phase

Cd'=(k_cd1*AP1+k_cd2*E2F*(K_i7/(K_i7+pRB))*(K_i8/(K_i8+pRBp))-k_com1*Cd*\

(Cdk4_tot-(Mdi+Md+Mdp27))+k_decom1*Mdi-V_dd*(Cd/(K_dd+Cd))-k_ddd*Cd)*eps

Mdi'=(k_com1*Cd*(Cdk4_tot-(Mdi+Md+Mdp27))-k_decom1*Mdi+V_m2d*(Md/(K_2d+Md))-\

V_m1d*(Mdi/(K_1d+Mdi)))*eps

Md'=(V_m1d*(Mdi/(K_1d+Mdi))-V_m2d*(Md/(K_2d+Md))-k_c1*Md*p27+k_c2*Mdp27)*eps

Mdp27'=(k_c1*Md*p27-k_c2*Mdp27)*eps

#Module Cyclin E/Cdk2: G1 phase and transition G1/S

Mce'=v_sce*K_ice^nce/(K_ice^nce+Bn^nce)-V_dmce*Mce/(K_dmce+Mce)

Ce'=(k_ce*E2F*(K_i9/(K_i9+pRB))*(K_i10/(K_i10+pRBp))+k_ce2*Mce-k_com2*Ce*\

(Cdk2_tot-(Mei+Me+Mep27+Mai+Ma+Map27))+k_decom2*Mei-V_de*(Skp2/(K_dceskp2+Skp2))*\

(Ce/(K_de+Ce))-k_dde*Ce)*eps

Mei'=(k_com2*Ce*(Cdk2_tot-(Mei+Me+Mep27+Mai+Ma+Map27))-k_decom2*Mei+V_m2e*(Wee1+i_b1)\

*(Me/(K_2e+Me))-V_m1e*Pe*(Mei/(K_1e+Mei)))*eps

Me'=(V_m1e*Pe*(Mei/(K_1e+Mei))-V_m2e*(Wee1+i_b1)*(Me/(K_2e+Me))-k_c3*Me*p27+k_c4*Mep27)*eps

Skp2'=(v_sskp2-V_dskp2*(Skp2/(K_dskp2+Skp2))*(Cdh1a/(K_cdh1+Cdh1a))-k_ddskp2*Skp2)*eps

Mep27'=(k_c3*Me*p27-k_c4*Mep27)*eps

Pei'=(v_spei+V_6e*(x_e1+x_e2*Chk1)*(Pe/(K_6e+Pe))-V_m5e*(Me+a_e)*(Pei/(K_5e+Pei))\

-k_dpei*Pei)*eps

Pe'=(V_m5e*(Me+a_e)*(Pei/(K_5e+Pei))-V_6e*(x_e1+x_e2*Chk1)*(Pe/(K_6e+Pe))-k_dpe*Pe)*eps

# Module Cyclin A/Cdk2 : S phase and transition S/G2

Ca'=(k_ca*E2F*(K_i11/(K_i11+pRB))*(K_i12/(K_i12+pRBp))-k_com3*Ca*\

(Cdk2_tot-(Mei+Me+Mep27+Mai+Ma+Map27))+\

k_decom3*Mai-V_da*(Ca/(K_da+Ca))*(Cdc20a/(K_acdc20+Cdc20a))-k_dda*Ca)*eps

Mai'=(k_com3*Ca*(Cdk2_tot-(Mei+Me+Mep27+Mai+Ma+Map27))-k_decom3*Mai+V_m2a*(Wee1+i_b2)\

*(Ma/(K_2a+Ma))-V_m1a*Pa*(Mai/(K_1a+Mai)))*eps

Ma'=(V_m1a*Pa*(Mai/(K_1a+Mai))-V_m2a*(Wee1+i_b2)*(Ma/(K_2a+Ma))-k_c5*Ma*p27+k_c6*Map27)*eps

Map27'=(k_c5*Ma*p27-k_c6*Map27)*eps

p27'=(v_s1p27+v_s2p27*E2F*(K_i13/(K_i13+pRB))*(K_i14/(K_i14+pRBp))-k_c1*Md*p27+k_c2*Mdp27\

-k_c3*Me*p27+k_c4*Mep27-k_c5*Ma*p27+k_c6*Map27\

-k_c7*Mb*p27+k_c8*Mbp27-V_1p27*Me*(p27/(K_1p27+p27))+V_2p27*(p27p/(K_2p27+p27p))\

-k_ddp27*p27)*eps

p27p'=(V_1p27*Me*(p27/(K_1p27+p27))-V_2p27*(p27p/(K_2p27+p27p))-V_dp27p*\

(Skp2/(K_dp27skp2+Skp2))*(p27p/(K_dp27p+p27p))-k_ddp27p*p27p)*eps

Cdh1i'=(V_2cdh1*(Cdh1a/(K_2cdh1+Cdh1a))*(Ma+Mb)-V_1cdh1*(Cdh1i/(K_1cdh1+Cdh1i))-\

k_dcdh1i*Cdh1i)*eps

Cdh1a'=(v_scdh1a+V_1cdh1*(Cdh1i/(K_1cdh1+Cdh1i))-V_2cdh1*(Cdh1a/(K_2cdh1+Cdh1a))*\

(Ma+Mb)-k_dcdh1a*Cdh1a)*eps

Pai'=(v_spai+V_6a*(x_a1+x_a2*Chk1)*(Pa/(K_6a+Pa))-V_m5a*(Ma+a_a)*(Pai/(K_5a+Pai))-\

k_dpai*Pai)*eps

Pa'=(V_m5a*(Ma+a_a)*(Pai/(K_5a+Pai))-V_6a*(x_a1+x_a2*Chk1)*(Pa/(K_6a+Pa))-k_dpa*Pa)*eps

# Module Cyclin B/Cdk1 : G2 phase and transition G2/M

Cb'=(v_cb-k_com4*Cb*(Cdk1_tot-(Mbi+Mb+Mbp27))+k_decom4*Mbi-V_db*(Cb/(K_db+Cb))*\

((Cdc20a/(K_dbcdc20+Cdc20a))+(Cdh1a/(K_dbcdh1+Cdh1a)))-k_ddb*Cb)*eps

Mbi'=(k_com4*Cb*(Cdk1_tot-(Mbi+Mb+Mbp27))-k_decom4*Mbi+V_m2b*(Wee1+i_b3)*(Mb/(K_2b+Mb))\

-V_m1b*Pb*(Mbi/(K_1b+Mbi)))*eps

Mb'=(V_m1b*Pb*(Mbi/(K_1b+Mbi))-V_m2b*(Wee1+i_b3)*(Mb/(K_2b+Mb))-k_c7*Mb*p27+k_c8*Mbp27)*eps

Mbp27'=(k_c7*Mb*p27-k_c8*Mbp27)*eps

Cdc20i'=(v_scdc20i-V_m3b*Mb*(Cdc20i/(K_3b+Cdc20i))+V_m4b*(Cdc20a/(K_4b+Cdc20a))\

-k_dcdc20i*Cdc20i)*eps

Cdc20a'=(V_m3b*Mb*(Cdc20i/(K_3b+Cdc20i))-V_m4b*(Cdc20a/(K_4b+Cdc20a))-k_dcdc20a*Cdc20a)*eps

Pbi'=(v_spbi+V_6b*(x_b1+x_b2*Chk1)*(Pb/(K_6b+Pb))-V_m5b*(Mb+a_b)*\

(Pbi/(K_5b+Pbi))-k_dpbi*Pbi)*eps

Pb'=(V_m5b*(Mb+a_b)*(Pbi/(K_5b+Pbi))-V_6b*(x_b1+x_b2*Chk1)*(Pb/(K_6b+Pb))-k_dpb*Pb)*eps

#coupling

Mw'=v_swee1+v_sw*Bn^nmw/(K_aw^nmw+Bn^nmw)-V_dmw*Mw/(K_dmw+Mw)

Wee1'=(k_sw*Mw-V_m7b*(Mb+i_b)*(Wee1/(K_7b+Wee1))+V_m8b*(Wee1p/(K_8b+Wee1p))-k_dwee1*Wee1)*eps

Wee1p'=(V_m7b*(Mb+i_b)*(Wee1/(K_7b+Wee1))-V_m8b*(Wee1p/(K_8b+Wee1p))-k_dwee1p*Wee1p)*eps

#parameters

par k1_clock=0.8,k2_clock=0.4,k3_clock=0.8,k4_clock=0.4,k5=0.8,k6=0.4,k7=1,k8=0.2,k9=0.8,k10=0.4,\

K_AP=0.6,K_AC=0.6,K_AR=0.6,K_IB=1,\

k_dmb=0.02,k_dmc=0.02,k_dmp=0.02,k_dmr=0.02,k_dn=0.02,k_dnc=0.02,\

K_d=0.3,K_dp=0.1,K_p=1.006,K_mB=0.4,K_mC=0.4,K_mP=0.3,K_mR=0.4,k_sB=0.32,\

k_sC=3.2,k_sP=1.2,k_sR=1.7,m=2,h=2,n=2,\

V_1B=1.4,V_1C=1.2,V_1P=9.6,V_1PC=2.4,V_2B=0.2,V_2C=0.2,V_2P=0.6,V_2PC=0.2,\

V_3B=1.4,V_3PC=2.4,V_4B=0.4,V_4PC=0.2,V_phos=0.4,v_dBC=3,v_dBN=3,v_dCC=1.4,\

v_dIN=1.6,v_dPC=3.4,v_dPCC=1.4,v_dPCN=1.4,v_dRC=4.4,v_dRN=0.8,v_mB=1.3,\

v_mC=2.0,v_mP=2.2,v_mR=1.6,v_sB=1.8,\

v_sC=2.2,v_sP=2.4,v_sR=1.6

#Cell cycle

par Chk1=0,u=1

par GF=1,K_agf=0.1,k_dap1=0.15,eps=22.2,v_sap1=1

par k_de2f=0.002,k_de2fp=1.1,k_dprb=0.01,k_dprbp=0.06,k_dprbpp=0.04

par k_pc1=0.05,k_pc2=0.5,k_pc3=0.025,k_pc4=0.5,K1=0.1,K2=0.1,K3=0.1

par K4=0.1,V1=2.2,V2=2,V3=1,V4=2,K_1e2f=5,K_2e2f=5,V_1e2f=4

par V_2e2f=0.75,v_se2f=0.15,v_sprb=0.8

par Cdk4_tot=1.5,K_i7=0.1,K_i8=2,k_cd1=0.4,k_cd2=0.005,k_decom1=0.1

par k_com1=0.175,k_c1=0.15,k_c2=0.05,k_ddd=0.005,K_dd=0.1,K_1d=0.1,K_2d=0.1

par V_dd=5,V_m1d=1,V_m2d=0.2

par a_e=0.25,Cdk2_tot=2,i_b1=0.5,K_i9=0.1,K_i10=2,k_ce=0.29,k_c3=0.2

par k_c4=0.1,k_decom2=0.1,k_com2=0.2,k_dde=0.005,k_ddskp2=0.005,k_dpe=0.075

par k_dpei=0.15,K_de=0.1,K_dceskp2=2,K_dskp2=0.5,K_cdh1=0.4,K_1e=0.1

par K_2e=0.1,K_5e=0.1,K_6e=0.1,V_de=3,V_dskp2=1.1,V_m1e=2,V_m2e=1.4,V_m5e=5

par V_6e=0.8,v_spei=0.13,v_sskp2=0.15,x_e1=1,x_e2=1

par a_a=0.2,i_b2=0.5,K_i11=0.1,K_i12=2,K_i13=0.1,K_i14=2,k_ca=0.0375

par k_decom3=0.1,k_com3=0.2,k_c5=0.15,k_c6=0.125,k_dda=0.005,k_ddp27=0.06

par k_ddp27p=0.01,k_dcdh1a=0.1,k_dcdh1i=0.2,k_dpa=0.075,k_dpai=0.15,K_da=1.1

par K_dp27p=0.1,K_dp27skp2=0.1,K_acdc20=2,K_1a=0.1,K_2a=0.1,K_1cdh1=0.01

par K_2cdh1=0.01,K_5a=0.1,K_6a=0.1,K_1p27=0.5,K_2p27=0.5,V_dp27p=5,V_da=2.5

par V_m1a=2,V_m2a=1.85,V_m5a=4,V_6a=1,v_scdh1a=0.11,v_spai=0.105

par v_s1p27=0.8,v_s2p27=0.1,V_1cdh1=1.25,V_2cdh1=8,V_1p27=100,V_2p27=0.1

par x_a1=1,x_a2=1

par a_b=0.11,Cdk1_tot=0.5,i_b=0.75,i_b3=0.5,k_c7=0.12,k_c8=0.2

par k_decom4=0.1,k_com4=0.25,k_dcdc20a=0.05,k_dcdc20i=0.14,k_ddb=0.005

par k_dpb=0.1,k_dpbi=0.2,k_dwee1=0.1,k_dwee1p=0.2,K_db=0.005,K_dbcdc20=0.2,K_dbcdh1=0.1

par k_sw=5,K_1b=0.1,K_2b=0.1,K_3b=0.1,K_4b=0.1,K_5b=0.1,K_6b=0.1,K_7b=0.1

par K_8b=0.1,v_cb=0.055,V_db=0.06,V_m1b=3.9,V_m2b=2.1,v_scdc20i=0.1,V_m3b=8,V_m4b=0.7

par V_m5b=5,V_6b=1,V_m7b=1.2,V_m8b=1,v_spbi=0.12,x_b1=1,x_b2=1

#coupling

par v_sw=0,v_swee1=0.0117,nmw=4,K_aw=2,V_dmw=0.5,K_dmw=0.5

par v_sce=0.06,K_ice=1,V_dmce=0.5,K_dmce=0.5,nce=4,k_ce2=5

#some initial conditions

init Mp=4.0342, Mc=4.6187, Mbmal=8.4909, Pc=0.02396, Cc=327.7673,\

Pcp=0.01385, Ccp=0.76132, PCc=3.9844, PCn=1.0813, PCcp=5.3548

init PCnp=0.62622, Bc=3.3185, Bcp=0.14085, Bn=1.7053, Bnp=0.09056,\

In=1.6584, Mr=2.6716, Rc=1.5322, Rn=1.1182

#cell cycle

init AP1=6.0606, pRB=1.4878, pRBc1=2.2087, pRBp=12.227, pRBc2=9.0762, pRBpp=0.05742, E2F=14.9737, E2Fp=0.00819

init Cd=0.09413, Mdi=0.02283, Md=1.3177, Mdp27=0.02085

init Mce=0.00645, Ce=0.00278, Mei=0.02898, Me=0.73755, Skp2=17.427, Mep27=0.00825, Pei=0.02175, Pe=1.6906

init Ca=0.00438, Mai=0.00979, Ma=0.00343, Map27=2.1468E-5, p27=0.0056, p27p=0.01954, Cdh1i=0.54659, Cdh1a=0.00675, Pai=0.59338, Pa=0.21284,

init Cb=0.2077, Mbi=0.03326, Mb=0.41118, Mbp27=0.00137, Cdc20i=0.0301, Cdc20a=1.9169, Pbi=0.06449, Pb=1.0719, Mw=0.01198, Wee1=0.11797

init Wee1p=0.2403

@ bounds=10000,total=1500,Method=Qualst.RK4,dt=0.01

@ runnow=1

done

**9.3 Coupling the cell cycle to circadian clock via both Wee1 and cyclin E**

Mp'=v_sP*Bn^n/(K_AP^n+Bn^n)-v_mP*Mp/(K_mP+Mp)-k_dmp*Mp

Mc'=v_sC*Bn^n/(K_AC^n+Bn^n)-v_mC*Mc/(K_mC+Mc)-k_dmc*Mc

Mbmal'=v_sB*K_IB^m/(K_IB^m+Rn^m)-v_mB*Mbmal/(K_mB+Mbmal)-k_dmb*Mbmal

Pc'=k_sP*Mp-V_1P*Pc/(K_p+Pc)+V_2P*Pcp/(K_dp+Pcp)+k4_clock*PCc-k3_clock*Pc*Cc-k_dn*Pc

Cc'=k_sC*Mc-V_1C*Cc/(K_p+Cc)+V_2C*Ccp/(K_dp+Ccp)+k4_clock*PCc-k3_clock*Pc*Cc-k_dnc*Cc

Pcp'=V_1P*Pc/(K_p+Pc)-V_2P*Pcp/(K_dp+Pcp)-v_dPC*Pcp/(K_d+Pcp)-k_dn*Pcp

Ccp'=V_1C*Cc/(K_p+Cc)-V_2C*Ccp/(K_dp+Ccp)-v_dCC*Ccp/(K_d+Ccp)-k_dn*Ccp

PCc'=-V_1PC*PCc/(K_p+PCc)+V_2PC*PCcp/(K_dp+PCcp)-k4_clock*PCc+k3_clock*Pc*Cc+\

k2_clock*PCn-k1_clock*PCc-k_dn*PCc

PCn'=-V_3PC*PCn/(K_p+PCn)+V_4PC*PCnp/(K_dp+PCnp)-k2_clock*PCn+k1_clock*PCc-k7*Bn\

*PCn+k8*In-k_dn*PCn

PCcp'=V_1PC*PCc/(K_p+PCc)-V_2PC*PCcp/(K_dp+PCcp)-v_dPCC*PCcp/(K_d+PCcp)-k_dn*PCcp

PCnp'=V_3PC*PCn/(K_p+PCn)-V_4PC*PCnp/(K_dp+PCnp)-v_dPCN*PCnp/(K_d+PCnp)-k_dn*PCnp

Bc'=k_sB*Mbmal-V_1B*Bc/(K_p+Bc)+V_2B*Bcp/(K_dp+Bcp)-k5*Bc+k6*Bn-k_dn*Bc

Bcp'=V_1B*Bc/(K_p+Bc)-V_2B*Bcp/(K_dp+Bcp)-v_dBC*Bcp/(K_d+Bcp)-k_dn*Bcp

Bn'=-V_3B*Bn/(K_p+Bn)+V_4B*Bnp/(K_dp+Bnp)+k5*Bc-k6*Bn-k7*Bn*PCn+k8*In-k_dn*Bn

Bnp'=V_3B*Bn/(K_p+Bn)-V_4B*Bnp/(K_dp+Bnp)-v_dBN*Bnp/(K_d+Bnp)-k_dn*Bnp

In'=-k8*In+k7*Bn*PCn-v_dIN*In/(K_d+In)-k_dn*In

Mr'=v_sR*Bn^h/(K_AR^h+Bn^h)-v_mR*Mr/(K_mR+Mr)-k_dmr*Mr

Rc'=k_sR*Mr-k9*Rc+k10*Rn-v_dRC*Rc/(K_d+Rc)-k_dn*Rc

Rn'=k9*Rc-k10*Rn-v_dRN*Rn/(K_d+Rn)-k_dn*Rn

#cell cycle

AP1'=(v_sap1*(GF/(K_agf+GF))-k_dap1*AP1)*eps

pRB'=(v_sprb-k_pc1*pRB*E2F+k_pc2*pRBc1-V1*(pRB/(K1+pRB))*(Md+Mdp27)+V2*(pRBp/(K2+pRBp))\

-k_dprb*pRB)*eps

pRBc1'=(k_pc1*pRB*E2F-k_pc2*pRBc1)*eps

pRBp'=(V1*(pRB/(K1+pRB))*(Md+Mdp27)-V2*(pRBp/(K2+pRBp))-V3*(pRBp/(K3+pRBp))*Me+\

V4*(pRBpp/(K4+pRBpp))-k_pc3*pRBp*E2F+k_pc4*pRBc2-k_dprbp*pRBp)*eps

pRBc2'=(k_pc3*pRBp*E2F-k_pc4*pRBc2)*eps

pRBpp'=(V3*(pRBp/(K3+pRBp))*Me-V4*(pRBpp/(K4+pRBpp))-k_dprbpp*pRBpp)*eps

E2F'=(v_se2f-k_pc1*pRB*E2F+k_pc2*pRBc1-k_pc3*pRBp*E2F+k_pc4*pRBc2-V_1e2f*Ma*\

(E2F/(K_1e2f+E2F))+V_2e2f*(E2Fp/(K_2e2f+E2Fp))-k_de2f*E2F)*eps

E2Fp'=(V_1e2f*Ma*(E2F/(K_1e2f+E2F))-V_2e2f*(E2Fp/(K_2e2f+E2Fp))-k_de2fp*E2Fp)*eps

# Module Cyclin D/Cdk4-6 : G1 phase

Cd'=(k_cd1*AP1+k_cd2*E2F*(K_i7/(K_i7+pRB))*(K_i8/(K_i8+pRBp))-k_com1*Cd*\

(Cdk4_tot-(Mdi+Md+Mdp27))+k_decom1*Mdi-V_dd*(Cd/(K_dd+Cd))-k_ddd*Cd)*eps

Mdi'=(k_com1*Cd*(Cdk4_tot-(Mdi+Md+Mdp27))-k_decom1*Mdi+V_m2d*(Md/(K_2d+Md))-\

V_m1d*(Mdi/(K_1d+Mdi)))*eps

Md'=(V_m1d*(Mdi/(K_1d+Mdi))-V_m2d*(Md/(K_2d+Md))-k_c1*Md*p27+k_c2*Mdp27)*eps

Mdp27'=(k_c1*Md*p27-k_c2*Mdp27)*eps

#Module Cyclin E/Cdk2: G1 phase and transition G1/S

Mce'=u*v_sce*K_ice^nce/(K_ice^nce+Bn^nce)-V_dmce*Mce/(K_dmce+Mce)

Ce'=(k_ce*E2F*(K_i9/(K_i9+pRB))*(K_i10/(K_i10+pRBp))+k_ce2*Mce-k_com2*Ce*\

(Cdk2_tot-(Mei+Me+Mep27+Mai+Ma+Map27))+k_decom2*Mei-V_de*(Skp2/(K_dceskp2+Skp2))*\

(Ce/(K_de+Ce))-k_dde*Ce)*eps

Mei'=(k_com2*Ce*(Cdk2_tot-(Mei+Me+Mep27+Mai+Ma+Map27))-k_decom2*Mei+V_m2e*(Wee1+i_b1)\

*(Me/(K_2e+Me))-V_m1e*Pe*(Mei/(K_1e+Mei)))*eps

Me'=(V_m1e*Pe*(Mei/(K_1e+Mei))-V_m2e*(Wee1+i_b1)*(Me/(K_2e+Me))-k_c3*Me*p27+k_c4*Mep27)*eps

Skp2'=(v_sskp2-V_dskp2*(Skp2/(K_dskp2+Skp2))*(Cdh1a/(K_cdh1+Cdh1a))-k_ddskp2*Skp2)*eps

Mep27'=(k_c3*Me*p27-k_c4*Mep27)*eps

Pei'=(v_spei+V_6e*(x_e1+x_e2*Chk1)*(Pe/(K_6e+Pe))-V_m5e*(Me+a_e)*(Pei/(K_5e+Pei))\

-k_dpei*Pei)*eps

Pe'=(V_m5e*(Me+a_e)*(Pei/(K_5e+Pei))-V_6e*(x_e1+x_e2*Chk1)*(Pe/(K_6e+Pe))-k_dpe*Pe)*eps

# Module Cyclin A/Cdk2 : S phase and transition S/G2

Ca'=(k_ca*E2F*(K_i11/(K_i11+pRB))*(K_i12/(K_i12+pRBp))-k_com3*Ca*\

(Cdk2_tot-(Mei+Me+Mep27+Mai+Ma+Map27))+\

k_decom3*Mai-V_da*(Ca/(K_da+Ca))*(Cdc20a/(K_acdc20+Cdc20a))-k_dda*Ca)*eps

Mai'=(k_com3*Ca*(Cdk2_tot-(Mei+Me+Mep27+Mai+Ma+Map27))-k_decom3*Mai+V_m2a*(Wee1+i_b2)\

*(Ma/(K_2a+Ma))-V_m1a*Pa*(Mai/(K_1a+Mai)))*eps

Ma'=(V_m1a*Pa*(Mai/(K_1a+Mai))-V_m2a*(Wee1+i_b2)*(Ma/(K_2a+Ma))-k_c5*Ma*p27+k_c6*Map27)*eps

Map27'=(k_c5*Ma*p27-k_c6*Map27)*eps

p27'=(v_s1p27+v_s2p27*E2F*(K_i13/(K_i13+pRB))*(K_i14/(K_i14+pRBp))-k_c1*Md*p27+k_c2*Mdp27\

-k_c3*Me*p27+k_c4*Mep27-k_c5*Ma*p27+k_c6*Map27\

-k_c7*Mb*p27+k_c8*Mbp27-V_1p27*Me*(p27/(K_1p27+p27))+V_2p27*(p27p/(K_2p27+p27p))\

-k_ddp27*p27)*eps

p27p'=(V_1p27*Me*(p27/(K_1p27+p27))-V_2p27*(p27p/(K_2p27+p27p))-V_dp27p*\

(Skp2/(K_dp27skp2+Skp2))*(p27p/(K_dp27p+p27p))-k_ddp27p*p27p)*eps

Cdh1i'=(V_2cdh1*(Cdh1a/(K_2cdh1+Cdh1a))*(Ma+Mb)-V_1cdh1*(Cdh1i/(K_1cdh1+Cdh1i))-\

k_dcdh1i*Cdh1i)*eps

Cdh1a'=(v_scdh1a+V_1cdh1*(Cdh1i/(K_1cdh1+Cdh1i))-V_2cdh1*(Cdh1a/(K_2cdh1+Cdh1a))*\

(Ma+Mb)-k_dcdh1a*Cdh1a)*eps

Pai'=(v_spai+V_6a*(x_a1+x_a2*Chk1)*(Pa/(K_6a+Pa))-V_m5a*(Ma+a_a)*(Pai/(K_5a+Pai))-\

k_dpai*Pai)*eps

Pa'=(V_m5a*(Ma+a_a)*(Pai/(K_5a+Pai))-V_6a*(x_a1+x_a2*Chk1)*(Pa/(K_6a+Pa))-k_dpa*Pa)*eps

# Module Cyclin B/Cdk1 : G2 phase and transition G2/M

Cb'=(v_cb-k_com4*Cb*(Cdk1_tot-(Mbi+Mb+Mbp27))+k_decom4*Mbi-V_db*(Cb/(K_db+Cb))*\

((Cdc20a/(K_dbcdc20+Cdc20a))+(Cdh1a/(K_dbcdh1+Cdh1a)))-k_ddb*Cb)*eps

Mbi'=(k_com4*Cb*(Cdk1_tot-(Mbi+Mb+Mbp27))-k_decom4*Mbi+V_m2b*(Wee1+i_b3)*(Mb/(K_2b+Mb))\

-V_m1b*Pb*(Mbi/(K_1b+Mbi)))*eps

Mb'=(V_m1b*Pb*(Mbi/(K_1b+Mbi))-V_m2b*(Wee1+i_b3)*(Mb/(K_2b+Mb))-k_c7*Mb*p27+k_c8*Mbp27)*eps

Mbp27'=(k_c7*Mb*p27-k_c8*Mbp27)*eps

Cdc20i'=(v_scdc20i-V_m3b*Mb*(Cdc20i/(K_3b+Cdc20i))+V_m4b*(Cdc20a/(K_4b+Cdc20a))\

-k_dcdc20i*Cdc20i)*eps

Cdc20a'=(V_m3b*Mb*(Cdc20i/(K_3b+Cdc20i))-V_m4b*(Cdc20a/(K_4b+Cdc20a))-k_dcdc20a*Cdc20a)*eps

Pbi'=(v_spbi+V_6b*(x_b1+x_b2*Chk1)*(Pb/(K_6b+Pb))-V_m5b*(Mb+a_b)*\

(Pbi/(K_5b+Pbi))-k_dpbi*Pbi)*eps

Pb'=(V_m5b*(Mb+a_b)*(Pbi/(K_5b+Pbi))-V_6b*(x_b1+x_b2*Chk1)*(Pb/(K_6b+Pb))-k_dpb*Pb)*eps

#coupling

Mw'=v_swee1+u*v_sw*Bn^nmw/(K_aw^nmw+Bn^nmw)-V_dmw*Mw/(K_dmw+Mw)

Wee1'=(k_sw*Mw-V_m7b*(Mb+i_b)*(Wee1/(K_7b+Wee1))+V_m8b*(Wee1p/(K_8b+Wee1p))-k_dwee1*Wee1)*eps

Wee1p'=(V_m7b*(Mb+i_b)*(Wee1/(K_7b+Wee1))-V_m8b*(Wee1p/(K_8b+Wee1p))-k_dwee1p*Wee1p)*eps

#parameters

par k1_clock=0.8,k2_clock=0.4,k3_clock=0.8,k4_clock=0.4,k5=0.8,k6=0.4,k7=1,k8=0.2,k9=0.8,k10=0.4,\

K_AP=0.6,K_AC=0.6,K_AR=0.6,K_IB=1,\

k_dmb=0.02,k_dmc=0.02,k_dmp=0.02,k_dmr=0.02,k_dn=0.02,k_dnc=0.02,\

K_d=0.3,K_dp=0.1,K_p=1.006,K_mB=0.4,K_mC=0.4,K_mP=0.3,K_mR=0.4,k_sB=0.32,\

k_sC=3.2,k_sP=1.2,k_sR=1.7,m=2,h=2,n=2,\

V_1B=1.4,V_1C=1.2,V_1P=9.6,V_1PC=2.4,V_2B=0.2,V_2C=0.2,V_2P=0.6,V_2PC=0.2,\

V_3B=1.4,V_3PC=2.4,V_4B=0.4,V_4PC=0.2,V_phos=0.4,v_dBC=3,v_dBN=3,v_dCC=1.4,\

v_dIN=1.6,v_dPC=3.4,v_dPCC=1.4,v_dPCN=1.4,v_dRC=4.4,v_dRN=0.8,v_mB=1.3,\

v_mC=2.0,v_mP=2.2,v_mR=1.6,v_sB=1.8,\

v_sC=2.2,v_sP=2.4,v_sR=1.6

#Cell cycle

par Chk1=0,u=0.04

par GF=1,K_agf=0.1,k_dap1=0.15,eps=20.4,v_sap1=1

par k_de2f=0.002,k_de2fp=1.1,k_dprb=0.01,k_dprbp=0.06,k_dprbpp=0.04

par k_pc1=0.05,k_pc2=0.5,k_pc3=0.025,k_pc4=0.5,K1=0.1,K2=0.1,K3=0.1

par K4=0.1,V1=2.2,V2=2,V3=1,V4=2,K_1e2f=5,K_2e2f=5,V_1e2f=4

par V_2e2f=0.75,v_se2f=0.15,v_sprb=0.8

par Cdk4_tot=1.5,K_i7=0.1,K_i8=2,k_cd1=0.4,k_cd2=0.005,k_decom1=0.1

par k_com1=0.175,k_c1=0.15,k_c2=0.05,k_ddd=0.005,K_dd=0.1,K_1d=0.1,K_2d=0.1

par V_dd=5,V_m1d=1,V_m2d=0.2

par a_e=0.25,Cdk2_tot=2,i_b1=0.5,K_i9=0.1,K_i10=2,k_ce=0.29,k_c3=0.2

par k_c4=0.1,k_decom2=0.1,k_com2=0.2,k_dde=0.005,k_ddskp2=0.005,k_dpe=0.075

par k_dpei=0.15,K_de=0.1,K_dceskp2=2,K_dskp2=0.5,K_cdh1=0.4,K_1e=0.1

par K_2e=0.1,K_5e=0.1,K_6e=0.1,V_de=3,V_dskp2=1.1,V_m1e=2,V_m2e=1.4,V_m5e=5

par V_6e=0.8,v_spei=0.13,v_sskp2=0.15,x_e1=1,x_e2=1

par a_a=0.2,i_b2=0.5,K_i11=0.1,K_i12=2,K_i13=0.1,K_i14=2,k_ca=0.0375

par k_decom3=0.1,k_com3=0.2,k_c5=0.15,k_c6=0.125,k_dda=0.005,k_ddp27=0.06

par k_ddp27p=0.01,k_dcdh1a=0.1,k_dcdh1i=0.2,k_dpa=0.075,k_dpai=0.15,K_da=1.1

par K_dp27p=0.1,K_dp27skp2=0.1,K_acdc20=2,K_1a=0.1,K_2a=0.1,K_1cdh1=0.01

par K_2cdh1=0.01,K_5a=0.1,K_6a=0.1,K_1p27=0.5,K_2p27=0.5,V_dp27p=5,V_da=2.5

par V_m1a=2,V_m2a=1.85,V_m5a=4,V_6a=1,v_scdh1a=0.11,v_spai=0.105

par v_s1p27=0.8,v_s2p27=0.1,V_1cdh1=1.25,V_2cdh1=8,V_1p27=100,V_2p27=0.1

par x_a1=1,x_a2=1

par a_b=0.11,Cdk1_tot=0.5,i_b=0.75,i_b3=0.5,k_c7=0.12,k_c8=0.2

par k_decom4=0.1,k_com4=0.25,k_dcdc20a=0.05,k_dcdc20i=0.14,k_ddb=0.005

par k_dpb=0.1,k_dpbi=0.2,k_dwee1=0.1,k_dwee1p=0.2,K_db=0.005,K_dbcdc20=0.2,K_dbcdh1=0.1

par k_sw=5,K_1b=0.1,K_2b=0.1,K_3b=0.1,K_4b=0.1,K_5b=0.1,K_6b=0.1,K_7b=0.1

par K_8b=0.1,v_cb=0.055,V_db=0.06,V_m1b=3.9,V_m2b=2.1,v_scdc20i=0.1,V_m3b=8,V_m4b=0.7

par V_m5b=5,V_6b=1,V_m7b=1.2,V_m8b=1,v_spbi=0.12,x_b1=1,x_b2=1

#coupling

par v_sw=1,v_swee1=0.0117,nmw=4,K_aw=2,V_dmw=0.5,K_dmw=0.5

par v_sce=1,K_ice=1,V_dmce=0.5,K_dmce=0.5,nce=4,k_ce2=5

#some initial conditions

init Mp=1.0003, Mc=1.698, Mbmal=6.805, Pc=0.0055822, Cc=328.4629,\

Pcp=0.0031028, Ccp=0.76158, PCc=0.75418, PCn=0.2771, PCcp=5.8789

init PCnp=0.44121, Bc=1.9198, Bcp=0.1102, Bn=1.0386, Bnp=0.065823,\

In=0.070079, Mr=0.82908, Rc=0.12613, Rn=0.036569

#cell cycle

init AP1=6.0606, pRB=1.5921, pRBc1=0.20208, pRBp=13.5477, pRBc2=0.86082, pRBpp=0.091333, E2F=1.2949, E2Fp=0.11011

init Cd=0.094086, Mdi=0.022841, Md=1.3225, Mdp27=0.016087

init Mce=0.028122, Ce=0.006114, Mei=0.029935, Me=0.96505, Skp2=10.6707, Mep27=0.0081096, Pei=0.017005, Pe=1.6997

init Ca=0.0044513, Mai=0.051508, Ma=0.16472, Map27=0.00099041, p27=0.0042028, p27p=0.019275, Cdh1i=0.54806, Cdh1a=0.0037106, Pai=0.20488, Pa=1.0414

init Cb=1.0856, Mbi=0.033732, Mb=0.45296, Mbp27=0.0011308, Cdc20i=0.026537, Cdc20a=1.8263, Pbi=0.057179, Pb=1.0824, Mw=0.013442, Wee1=0.11901

init Wee1p=0.26963

@ bounds=10000,total=200,Method=Qualst.RK4

@ runnow=1

done
